# COVID-19 Modeling Outcome versus Reality in Sweden

**DOI:** 10.3390/v14081840

**Published:** 2022-08-22

**Authors:** Marcus Carlsson, Cecilia Söderberg-Nauclér

**Affiliations:** 1 Centre for Mathematical Sciences, Lund University, 22100 Lund, Sweden; 2 Department of Medicine, Solna, BioClinicum, Karolinska Institutet, 171 64 Solna, Sweden; 3 Department of Neurology, Karolinska University Hospital, 171 77 Stockholm, Sweden; 4 Department of Biosciences, InFLAMES Research Flagship Center, MediCity, University of Turku, 20500 Turku, Finland

**Keywords:** modeling, SARS-CoV-2, COVID-19, SIR, SEIR

## Abstract

It has been very difficult to predict the development of the COVID-19 pandemic based on mathematical models for the spread of infectious diseases, and due to major non-pharmacological interventions (NPIs), it is still unclear to what extent the models would have fit reality in a “do nothing” scenario. To shed light on this question, the case of Sweden during the time frame from autumn 2020 to spring 2021 is particularly interesting, since the NPIs were relatively minor and only marginally updated. We found that state of the art models are significantly overestimating the spread, unless we assume that social interactions significantly decrease continuously throughout the time frame, in a way that does not correlate well with Google-mobility data nor updates to the NPIs or public holidays. This leads to the question of whether modern SEIR-type mathematical models are unsuitable for modeling the spread of SARS-CoV-2 in the human population, or whether some particular feature of SARS-CoV-2 dampened the spread. We show that, by assuming a certain level of pre-immunity to SARS-CoV-2, we obtain an almost perfect data-fit, and discuss what factors could cause pre-immunity in the mathematical models. In this scenario, a form of herd-immunity under the given restrictions was reached twice (first against the Wuhan-strain and then against the alpha-strain), and the ultimate decline in cases was due to depletion of susceptibles rather than the vaccination campaign.

## 1. Introduction

### 1.1. Background

In March 2020, when it became clear that the COVID-19 outbreak was turning into a global pandemic, mathematical models were used to predict the magnitude of viral spread and health care needs, which had a major impact on public policy. For example, the Imperial College report No. 9 predicted an 81% hit-rate within a couple of months, in the worst case scenario [1], if the government did nothing to mitigate or suppress the pandemic. This led to a subsequent strategy shift towards suppression and eventually lock-down in the UK on 23 March 2020, as well as in many other countries world-wide. In sharp contrast, the Swedish government decided to keep the society relatively open and maintained relatively minor and rather fixed non-pharmacological interventions (NPIs) until the summer of 2021, when the majority of the population had been vaccinated. Sweden therefore provides an excellent example that can be used to test the validity of epidemiological mathematical models. Rather surprisingly, in the light of typical modeling outcomes, it is estimated that the total hit-rate in major Swedish cities was just above 20% in March 2021, one year after the onset of the pandemic [2] (Supplementary Material explains how to access the data in [2]). In this article, we analyze potential explanations for this drastic reduction.

**Figure 1:**
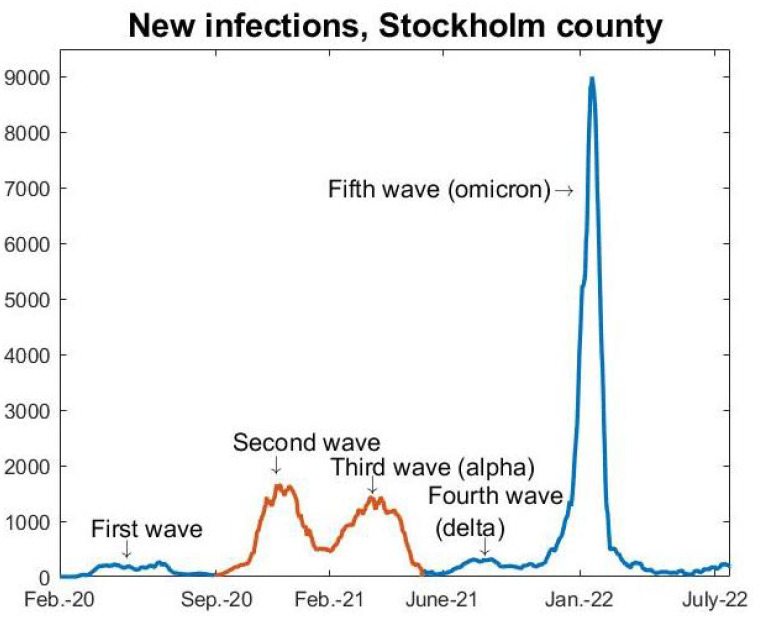
Full data series (weekly average) from the Swedish Public Health Agency. During first wave, only patients admitted to hospital were tested, so the data are very unreliable. General testing started in late May 2020. The red section indicates the time frame we focus on in this paper.

In April 2020, when seroprevalence measures indicated a hit rate of around 5% [2], we observed a decline in admissions to intensive care units (ICUs) that continued through May and led to very low new ICU-admissions during the summer of 2020, indicating that the viral spread in society was very low as well. This is also visible in Figure 1, displaying daily cases of COVID-19 in Stockholm County. Note that the data from the “first wave” is very unreliable since there was no large-scale public testing before May 2020.

When a major deadly epidemic hits, the society reacts in a way that is impossible to predict mathematically, sometimes referred to as “herd-protection”. Therefore, mathematical models are often grossly wrong in their predictions, as exemplified by previous potential disasters, such as various Ebola outbreaks [3,4]. This phenomenon, i.e., major voluntary reductions in social interactions, could, in theory, explain the difference between model outcome and reality also for SARS-CoV-2 in Sweden. However, the decline of the first wave happened despite the fact that schools were open and face-masks were not used. Even so, the seroprevalence among children aged 0–19 was very low, just above 6%, in June 2020 after the first wave [2]. It seemed difficult to envision such a development was the result of NPIs and voluntary measures alone, indicating that a part of the population could have a higher protection against contracting SARS-CoV-2, i.e., having some sort of pre-immunity.

### 1.2. Contributions

We use publicly available data from the Swedish Public Health Agency regarding incidence, variants, vaccination coverage and seroprevalence of SARS-CoV-2 in Stockholm County, Sweden, along with tailor-made mathematical models, in order to test these different explanations of what kept the spread at bay in Sweden. We base our model on an extension of a SEIR-model with age-stratification, developed by Britton et al. [5], which we update to include antibody waning and vaccination roll-out. For comparison with real data we chose the metropolitan area of Stockholm with about 2,400,000 inhabitants, since effects due to geographical isolation are likely negligible, compared with more scarcely populated areas elsewhere. We test the hypotheses: the observed fluctuations in cases are due to:

(1) Variations in NPIs and/or population behavior over time;
(2) Some form of protective pre-immunity and natural depletion of susceptibles.

Although the reality certainly could be a mixture of both, we find that the former hypothesis is unlikely to be the sole explanation, whereas we obtain a good data fit in the second hypothesis (which assumes that social contact patterns were constant during the time frame studied). In this scenario, our modeling implies that Sweden has reached herd immunity twice (given the mild restrictions), once to the Wuhan-strain in early December 2020, and thereafter to the alpha-strain in the beginning of April of 2021. Additionally, the effect of the vaccination scheme in the decline of the alpha-wave seen in May 2021 is, given this hypothesis, marginal.

In a separate study [6], we demonstrate that, from a modeling perspective, a number of population heterogeneities, such as variable susceptibility, variable activity patterns, as well as self-isolatory measures, seem to have an effect identical to assuming that a certain proportion of the population had a sterilizing pre-immunity. This “pre-immunity”, if it indeed existed, is thus likely a manifestation of a much more complex phenomenon than the name suggests, and to underline this we will refer to it as “artificial pre-immunity”. In other words, it is possible that including “artificial pre-immunity” in mathematical models is crucial for good model fit, even if sterilizing immunity against the virus, a priori to the disease, does not exist on an individual level. A more rigorous attempt at explaining what this artificial pre-immunity derives from is found in [7], which we also discuss to some extent in Section 5.3.

## 2. The COVID-19 Pandemic in Sweden

### 2.1. Choice of Time Frame

**Figure 2:**
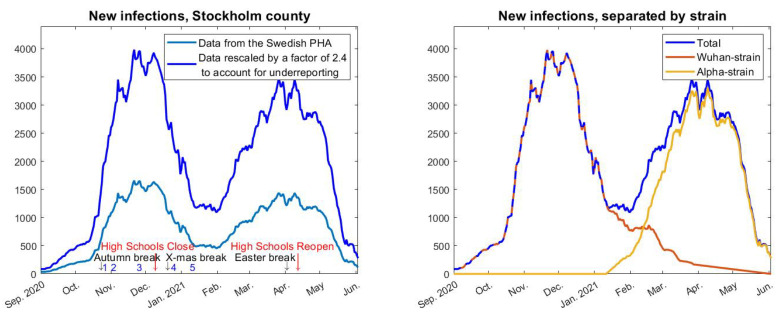
(**Left**): 7-day rolling average of new cases in Stockholm county, adjusted to match the reported increase in seroprevalence of antibodies against SARS-CoV-2. The numbers 1–5 correspond with the updates to the NPIs in Table 1. (**Right**): total cases separated in Wuhan-strain and Alpha-strain.

We removed the “first wave” from our study, for a number of reasons. First, testing was very unreliable, mainly limited to hospitalized patients only, and, hence, we have no data to compare the models with. Secondly, it is clear that major oscillations in social interactions took place during this period, and this can not be built into mathematical modeling in a reliable manner. Additionally, social interactions significantly drop during the Swedish summer vacations, which comes close to a soft lockdown. In September of 2020, when schools had reopened, society had largely returned to a “new normal”. At the time, most people were convinced there would be no more major waves in Sweden, and the chief epidemiologist continuously downplayed the situation, for example describing a 35% increase in new cases in Sweden as “a small cautious increase” [8]. During the autumn, the number of cases were never alarmingly high and the hospitals were coping with the stream of patients, and hence there was never a sense of panic. Indeed, at the peak of the second wave, seen as the first wave in Figure 2, the recommendations for the elderly to self-isolate were lifted, thus sending a message that the pandemic was under control to the population. It is crucial to underline that mathematical models work well, in theory, also during NPIs and voluntary social reductions, as long as these are kept fairly constant during the modeling time frame, and we believe that this was the case from September 2020 to May 2021. Another factor that speaks in favor of this time frame is that testing was widely available, so data from this period are reliable, (although needs to be adjusted to account for under-reporting of cases and asymptomatic infections). Furthermore, the fact that only two major strains are involved, the original Wuhan-strain and alpha, allows us to separate the amount of cases from each respective strain, which is key for modeling.

We do not include the delta and omicron waves in our study for a number of reasons, the key reason is that the majority of the population then was vaccinated, which significantly alters the model and moreover the data on seroprevalence no longer gives any information about how many had had COVID-19. Moreover, while it is clear that antibodies from both natural infection and the vaccine protected against delta, it is not clear to what degree, and, hence, it is not possible to perform any reliable mathematical modeling. Concerning the omicron-wave, it is known that neither antibodies from the vaccine nor natural infection gave a strong protection against contracting omicron, but to what extent did it give partial protection? Without a certain answer to these questions, further mathematical modeling becomes too speculative. Of course, the fact that there was no major delta wave but a substantial omicron wave is interesting, and will be discussed further in Section 5.2

Based on the above remarks, we argue that the time frame from September 2020 to May 2021 is optimal for mathematical modeling.

### 2.2. Separating the Data

Data for new cases in Stockholm County based on PCR-testing, which has been obtained from the Swedish Public Health Agency “Folkhälsomyndigheten” (see Supplementary Material for details) and averaged over a 7 day window, is displayed in Figure 1 and a zoom of the relevant time frame is displayed in Figure 2 (left). The most important thing to realize in order to understand the graph in Figure 2 is that what we see is two distinct waves superimposed. Thanks to available data on variants of concern collected by the Swedish Public Health Agency [9], we know what percentage is caused by the Wuhan strain and the variants of concern, respectively. The second wave seen in the figure (i.e., the third wave in reality) is almost entirely caused by the “British variant” B.1.1.7, also known as alpha. It also contained small portions of other variants of concern, P.1, B.1.167, and B.1.351, but those caused, at most, 6% of the cases (in the month of February) and even less in the last measurement, so for simplicity we shall refer to it as the alpha-wave, whereas we shall call the first wave (in Figure 2) the Wuhan-wave. The separation in cases due to the original “Wuhan-wave” and “alpha-wave” is displayed in Figure 2 (right).

### 2.3. The Swedish NPIs

Sweden provided a unique opportunity to test mathematical models for disease progression, in particular the densely populated metropolitan area of Stockholm with about 2.4 million inhabitants, due to the virtually constant NPIs. The only major change in these during the time frame Sept 2020 to May 2021 was the closing of high-schools (age-group 15–18 years) on 7 December 2020 and subsequent reopening on 1 April 2021. Bars and restaurants have been open but only with table-service and a maximum of 4 people at each table. In addition, a number of minor changes in the recommendations were made, which are collected in Table 1, (also marked in the timeline in Figure 2). It should be noted that public compliance with these restrictions was not ideal, for example less than one out of three was reported to follow the recommendation concerning facemasks in public transport, based on random testing performed by the Swedish Public Broadcasting company [10].

Neither these policy-updates nor any of the major holidays (Autumn, Christmas, Sport, and Easter school holidays), see Figure 2 (left), seems to have had any notable effect on the two waves. In particular, the Wuhan-wave was declining rapidly *before* the Christmas-break and subsequent mask-recommendations, and the alpha-strain had leveled out before the Easter break. It is also noteworthy that the *relaxations* 1–2 coincide with the time when the second wave looses momentum and levels out. Altogether this is an indication that neither holidays nor updates to the NPIs had a major impact on case numbers. Moreover, one has to bear in mind that the Swedish population had been informed by the Swedish Public Health Agency that frequent washing of hands and keeping 2 m distance between people are the key factors to limit the spread of the virus in society. Aerosol transmission was consistently denied by the Swedish Public Health Agency who maintained that droplet transmission was the main source of infections, which, for example, led to good ventilation not being part of the pandemic preventive measures in Sweden. Consequently, among those who did try to protect themselves, face-shields were about as common as face-masks, due to the wide-spread misconception that SARS-CoV-2 primarily spreads by infected individuals coughing and sneezing, which has been repeated over and over by spokesmen for the Swedish Public Health Agency. Since COVID-19 can spread by aerosols [11], particularly in indoor climate, we argue that the conditions for steady spread of COVID-19 were good throughout the time-period studied, in particular the cold and rainy months between October 2020 and May 2021 (April and May 2021 were unusually cold).

Furthermore, the first COVID-19 vaccinations started in Sweden after Christmas 2020, but people younger than 70+ were not offered vaccinations until early April 2021 (with the exception of special work groups, such as health care workers). As the elderly are not main drivers of the epidemic, and protective antibody levels take several weeks to develop, vaccinations most likely had limited effect on case numbers during the time frame in question. (Given the second hypothesis, we prove this last claim in Section 4.2.)

### 2.4. Under-Reporting and Seroprevalence Levels

Data on daily cases is difficult to use since there is always an unknown proportion of cases that do not get tested. To account for this, we have corrected the magnitude so that it fits with data from serological testing, which is a more stable indicator of total amount of infections, since the majority who have had COVID-19 go on to develop antibodies [12]. According to the Swedish Public Health Agency [2] (see also Supplementary Material), in early June 2020 the seroprevalence of antibodies against SARS-CoV-2 was 11.2%, which dropped to 9.8% mid-October 2020. Since this period was characterized by a very low prevalence of COVID-19, we assume that the drop is due to antibody waning. Interpolating between these data points, we estimate the seroprevalence at 10% in early September, the beginning of our time-line. The seroprevalence then rose to 22.8% in early March 2021 [2], at the time of the onset of the alpha-wave. Based on this, we estimate that there are 2.4 actual cases of COVID-19 for every reported case. The graph in Figure 2 has been amplified accordingly.

In the serological measurements performed by the Swedish Public Health Agency it is not possible to separate between antibodies caused by the virus and the vaccine, so the measurement from March 2021 is the last that gives an indication of how many had had COVID-19 at the given time. However, children under the age of 12 were not vaccinated in Sweden, and in Table 5 of [2] data from this age-group are displayed separately, indicating that the seroprevalence rose to just below 30% after the alpha wave, and remained at that level throughout the delta surge, which was very mild in Sweden. The seroprevalence then abruptly jumps to above 70% in March 2022, following the omicron-wave.

## 3. Methods

In this section, we briefly describe key ingredients in our modeling and processing of the data, as well as the underlying assumptions. A fuller description is found in Appendix A.

### 3.1. Choice of Model

We build our model upon the SEIR-model with age-stratification which was developed by Britton et al. [5]. This is a rather standard compartmental model, with 6 age-compartments for each category, Susceptible, Exposed, Infective, and Recovered, making 24 compartments in total. The flow of people between these compartments is governed by a system of ordinary differential equations, which is the standard way of building mathematical epidemiological models, see, e.g., [13] or [14].

We include age-stratification since it is one heterogeneity for which one can find realistic parameter values, and it allows us include the vaccination roll-out (which started among the elderly and gradually was offered to younger age-groups, see Appendix A.3 for details). For contact intensities between various age-groups we rely on the pre-pandemic study [15] but we updated the numbers to reflect Swedish demographics, along with a relatively higher reduction in contacts among the elderly, who were encouraged to self-isolate (see Appendix A.2 for details). Finally, to incorporate antibody waning we also have a flow of people moving from the recovered group to the susceptible group. We assume that antibodies for either the alpha or Wuhan strain gave protection against reinfection with a half-life of 16 months, (following [16] and also less precise observations in [17]). We implemented the code in MatLab, which can be downloaded from https://github.com/Marcus-Carlsson/Covid-modeling (accessed on 10 August 2022), and the details are further described in Appendix A.

**Figure 3:**
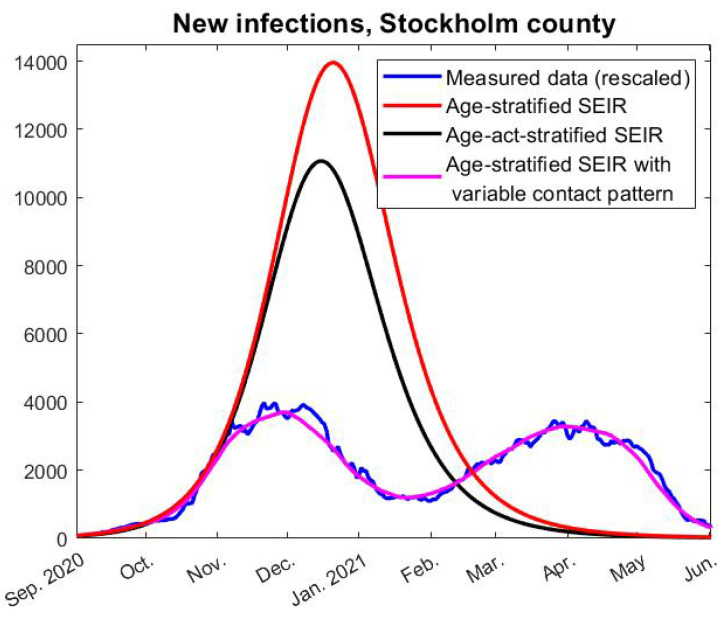
Comparison between real data and model outcome for the age-stratified SEIR and age-activity-stratified SEIR based on [5], which assumes that contact patterns are stable over time. The pink graph is produced with variable social interactions (over time), as described further in Section 4.1.

In Figure 3, we see a typical model outcome (red curve), where the parameters have been chosen to match the time series during the first two months September–October 2020. As is clear to see, it fits the data very well in those months, but in November the model curve continues to rise drastically. If this model had been used for policy decisions, then clearly a lock-down would have been called for, but no lock-down was ever imposed and some restrictions were even lifted, as explained earlier. Despite this, the measured case numbers start to level out and then sharply decay, and there is no indication of a resurge of cases before the introduction of the more contagious alpha-variant. The key research question of this paper is what caused this decay and why the model data is off (by a factor of around 3 compared to measured data). Are SEIR-type models simply poor for pandemic predictions, or is there something about human behavior or the virus itself which does not correspond to how the model is built?

We stress that nothing in the model we use is accelerating the spread compared with other models (in fact, Britton et al. developed it in an attempt to modify standard SEIR in order to *dampen* the spread). They also made a model that takes variability in the social activity level into account and demonstrated that such variations have a further damping effect on model curves. However, it is clearly impossible to measure such variations in the social activity level, and, hence, we cannot find realistic parameters for applying this model to a real society, wherefore we have built our model on the simpler age-stratified version. Nevertheless, we have also tested this model with the same parameters as in [5], and found that this does not dampen the model curves enough to alter any of the conclusions of this paper, even if the contrast between reality and model data becomes somewhat less stark, see Figure 3, (black curve).

Furthermore, the model we use is very similar to the one developed by a well renowned Swedish modeling team [18], as well as other models published for example by the Imperial College Team [19], in terms of the dynamics between the major groups Susceptible, Exposed, and Infected. These models also tend to drastically overestimate the spread. Indeed, ref. [18] predicts a cumulative number of infected people of around 30% after the first wave. The authors arrive at the “low” value of 30%, from a modeling perspective, due to assuming a 56% decrease in contacts among people of age 0–59 and a 98% reduction among those aged 60–79 (this is for scenario d), which accurately fitted ICU-occupancy and death, see Figure 2b in [18]. Given that many people in the latter age group are still working (average retirement age was 65 in Sweden 2021), an overall reduction of 98% in contacts seems somewhat unrealistic. More realistic reduction numbers, thus, give cumulative infection numbers above 30%, when the actual figure was around 10% (as we saw in Section 2.4), demonstrating the tendency of compartmental models to overestimate the spread, compared to what happened in reality (in Sweden).

Along the same lines, the famous Report 9 by the Imperial College [1] predicted a total number of 81% infected in a “do-nothing” scenario, based on a more advanced so called “agent-based model” that also treats household-contacts separately. According to Table 3 in the report [1], the number of deaths and peak ICU capacity can be reduced by 50% and 81%, respectively, in the most effective NPI-scenario, which certainly goes beyond what was implemented in Sweden. However, if these figures are directly adjusted to apply for Stockholm County, the predictions using the most severe restrictions still overestimate the actual numbers by a factor of roughly 4 (deaths) and 10 (ICU) (comparing with data from February 2021).

We remark that non-compartmental mathematical models, as found, e.g., in [20,21], have been more successful at predicting the pandemic in the short term, but we will not discuss this type of model further here.

### 3.2. Artificial Pre-Immunity and Population Heterogeneities

Before we introduce how to model with pre-immunity, we need to discuss what this actually means. Pre-immunity, as well as immunity developed after an infection, is not a binary variable that either gives a 100% protection (so-called “sterilizing immunity”) or none at all. In the separate study [6], we show that there is practically no difference mathematically between an advanced model taking variable susceptibility into account, and a more naive model which simply stipulates that a certain proportion of the population has sterilizing immunity. Hence, the idea of a binary pre-immunity works well on a macro-level, as long as one is aware that this is a simplification that does not apply on a micro-level. We remark that a number of different factors, such as genetic, cross-reactive immunity, and innate immunity, have been shown to provide variation in susceptibility [22,23,24]. In addition, whether a person gets infected or not upon exposure to SARS-CoV-2 clearly depends on the dosage as well as the time of exposure.

To complicate things further, a fraction of the population went into various degrees of self-isolation, and clearly it is impossible to distinguish such individuals from individuals with high-levels of natural defense against contracting SARS-CoV-2. However, it is shown numerically in [6] that such variations are also manifested as an artificial level of sterilizing immunity. In practice, this means, for example, that the black curve in Figure 3, produced by a model with 72 compartments (Age-Act-SEIR), could be obtained easily from the simpler 24-compartment Age-SEIR model, upon including artificial sterilizing immunity. Therefore, we have chosen to build our model on the simpler Age-SEIR-model while including a level of sterilizing immunity. This is a mathematical modeling simplification that comprises a number of complex factors, and it is impossible to know how much of this immunity comes from actual variations in susceptibility compared with variations in social interaction patterns. To underscore this, we have coined the term “Artificial Sterilizing Immunity” [6], but since this paper concerns the period when SARS-CoV-2 was relatively new, artificial sterilizing pre-immunity, or artificial pre-immunity for short, seems more accurate.

### 3.3. The Hypotheses Posed Mathematically

In order to transform the two hypotheses into mathematical language, we now describe in broad terms the key features of our model. Somewhat simplified, all mathematical models for disease progression rely on some refinement of the formula

(1) Newcases=a·β·i·s,

where *a* denotes the amount of daily contacts between individuals, β the probability that such a contact leads to disease transmission, *i* is the prevalence and *s* is the fraction of the population that are susceptible to the virus. Clearly *i* and *s* depend on time, but we shall also consider *a* and β as time dependent, to account for variations in social activity during the pandemic and the fact that the alpha strain was more contagious than the original strain.

In mathematical terms, Hypothesis 1 amounts to starting our model with s(1)=0.9 (i.e., 90% are susceptible on day 1, since 10% already had antibodies at the start of the timeline) and letting the amount of contacts vary with time. More precisely, we postulate that a(t)=a·(1−f(t)/100) where *a* is a constant chosen so that f(t)≈0 in September. In words, *f* describes the percentage-wise change in amount of contacts, comparing with September 2020 as a “normal month”.

Hypothesis 2, on the other hand, amounts to keeping the daily amount of contacts fixed, so a(t) is a constant, and instead allowing the initial value of s(1) to be substantially less than 0.9, to account for artificial pre-immunity. Since our model keeps track of infections caused by alpha and Wuhan-strain separately, we give the two strains individual β-values, as well as individual levels of artificial pre-immunity, since a natural protection against the original strain may be less effective against other variants.

## 4. Results

### 4.1. Hypothesis 1: Human Behavior

Recall that *f* describes the percentage-wise change in amount of contacts, as a function of time, comparing with September 2020 as a “normal month”. Given any fixed choice of *f*, our model produces a curve for the incidence which we can compare with measured data. We wish to underline that, in our modeling, we continuously remove vaccinated people (after the first shot) from the susceptible group to the recovered group, so *f* describes the updates in contact frequency with the vaccination roll-out already accounted for. Our model also takes into account, by default, that a majority of the elderly were self-isolating, so *f* really describes how the contact pattern in the remaining age-groups need to fluctuate. For further details we refer to Appendix A, in particular Appendix A.6.

**Figure 4:**
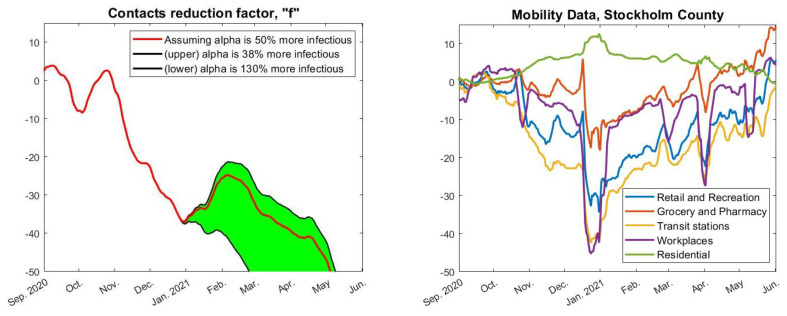
(**Left**): relative change in daily contacts needed to obtain a good fit with real data. The green area accounts for uncertainty about how much more contagious the alpha-strain was. The red central curve assumes 50% more contagious (following [16]), and the (almost perfect) data fit in Figure 3 (pink curve) is computed based on this. (**Right**): actual reduction in mobility pattern according to Google Mobility Data. Notice that the y-axis is the same in both the left and the right image, and that one would expect to see similar curves given that Hypothesis 1 holds.

In order to find an *f* so that the incidence-curve fits well with measured data, we also need to know how much more contagious the alpha-strain was compared with the original Wuhan-strain. In [25], they estimate this factor as between 38% and 130% more contagious (95% confidence interval), but this estimate comes with several uncertainties. If we suppose for the moment that it was 50% more contagious, then the red curve in Figure 4 describes how *f* (the relative change in social contacts) needs to fluctuate in order to obtain the data-fit seen by the pink graph in Figure 3. Hence, in order to match real data, we see that the contact frequency needs to continuously drop (except for a small increase in January) until it reaches levels around 50% lower in May 2021, compared with September 2020. This is clearly not realistic. On the contrary, May 2021 was characterized by a sense of relief as the case numbers were dropping, which officially was explained by the success of the vaccination campaign.

Of course, the less contagious alpha is, the less reduction in behavior is needed to keep the incidence down. This is the reason for the green shaded area, the upper boundary corresponds to assuming that alpha is only 38% more contagious, the lower by assuming it is 130% more contagious. As is plain to see, even in the 38% scenario, the fluctuations in *f* are not realistic.

To provide objective support for this affirmation, to the right in Figure 4 we see the actual behavior fluctuations according to Google Mobility Data. The data have been averaged weekly and adjusted so that the mean in September 2020 is 0. In order for the Wuhan-strain wave to turn around, as it did in November to mid-December 2020, the left image indicates that we need a reduction in *f* of around 30%. However, although Google Mobility Data does go down over the same period, it does so more modestly. For example “workplaces”, believed to be a key location for transmission, sees a reduction of around 10% in the relevant timespan, just before Christmas. Note, also, that the major fluctuations around the Christmas and Easter holidays, which are clearly visible in the Google-mobility graph, leave no clear bearing on the incidence, which is strange if human behavioral changes are the key factor for variations in incidence.

Moving to the second wave, the left image in Figure 4 indicates that we need another substantial reduction in human contacts from the month of February to May 2021, but, in stark contrast, throughout this period all relevant curves in the Google-mobility graph are going up. Let us again stress that, at this point, the seroprevalence measurements among children (as well as the data in Figure 2) indicates a total hit-rate of around 30% in June 2021 (Section 2.4), and that the incidence during autumn 2021 was kept low throughout the delta-surge (see Figure 1), even among unvaccinated children. Indeed, the September 2021 measurement in the groups 0–11, that were not given any vaccine but attended school as normal, had an estimated 28.4% seroprevalence, whereas the measurement in early December 2021 (after the delta surge) was 29.6% [2] (these data are easily accessible in Supplementary Material). This seems more to indicate that some sort of herd-immunity had been reached, and overall these observations cast doubt over Hypothesis 1 and indicate that other factors than human updates in mobility are needed in order to explain the two waves in Figure 2.

**Figure 5:**
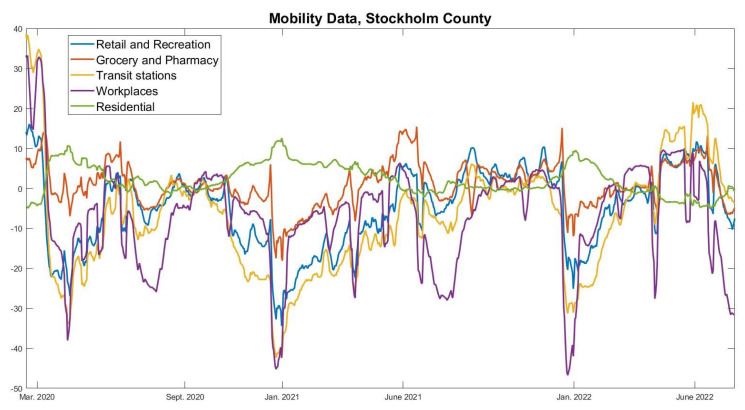
Google-mobility data over the full pandemic time-frame, preprocessed as in Figure 4.

To further underline this point, the Google-mobility data over the entire pandemic is displayed in Figure 5. Clearly, in March–April 2020 there is a significant reduction in attendance to work and transit stations. Beyond this point, the annual fluctuations are fairly similar, indicating that people had adapted a “new normal” that they kept throughout the pandemic. Although we see recurrent significant oscillations, particularly due to summer and Christmas holidays, no significant fluctuations in behavior can be coupled with the rise and fall of new cases seen in Figure 1. Overall, this coincides with the authors personal observations, i.e., that while people updated their behavior during the pandemic, they did *not* continuously change social patterns depending on whether the viral levels were high or not.

However, in this case, the SEIR-type epidemiological models should in theory work, but no such model will predict herd-immunity at around 30% for a virus with the characteristics of SARS-CoV-2. Thus, we are left with two possible options to explain what is seen in Figure 3; either the models of SEIR-type are not useful, or some unknown factor was dampening the viral spread in society.

### 4.2. Hypothesis 2: Depletion of Susceptibles

We now update the model so that a(t), the human contact rate, is constant throughout the modeling time-span, but, instead, we include a certain level of artificial pre-immunity, which we choose manually in order to obtain a good fit with measured data. As discussed earlier, if variable susceptibility on an individual level exists, it will manifest itself as (artificial) pre-immunity on a macroscopic level [6]. Thus, if a certain level of pre-immunity, say 50%, gives a good fit between model and reality, it does not mean that 50% are immune to SARS-CoV-2. However, a certain level of protection against the Wuhan-strain may work less well against the alpha-strain, and, hence, there is no reason to assume that the level of artificial pre-immunity would be the same against the two strains (just as we assume that they have different transmission probabilities $\beta_{Wuhan}$ and $\beta_{alpha}$).

**Figure 6:**
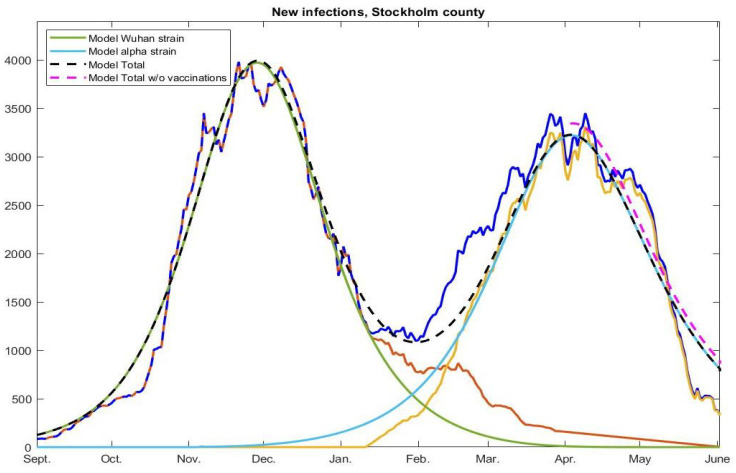
Actual and modeled curves using artificial pre-immunity. The brown-orange, yellow, and blue curves display measured cases, separated into Wuhan-strain, alpha-strain, and total, respectively, same as in Figure 2 (so reweighted as before). The green, light-blue, and dashed black display corresponding curves for our model. Finally, the pink dashed curve shows total model output in the absence of vaccinations (only displayed during April–May 2021). Clearly, if this model is correct, the vaccination campaign had virtually no part in the final demise during May 2021, contrary to popular belief.

The model output, shown in Figure 6, uses a 65% artificial pre-immunity against the Wuhan strain and a 56% artificial pre-immunity against alpha, in addition to the 10% natural immunity acquired before September 2020. The blue, light brown, and yellow represent the measured incidence (total, Wuhan, alpha) just as in Figure 2. The green color shows the model-incidence due to the Wuhan strain and light blue represents the alpha strain, whereas dashed brown represent the total model-incidence. As is plain to see, the model gives a very accurate fit.

For the Wuhan-strain wave (green), we have $R_{e}\approx 1.4$ in early September 2020. To obtain a good fit for the subsequent alpha-wave, we adjust the artificial pre-immunity down from 65% to 56% and leave the transmission probability unchanged. This would mean that what made alpha dominant was not a superior spreading capacity among those already susceptible to the Wuhan strain, but rather that it mutated in a way that it could get around some of the pre-immunity. For the blue model curve (for alpha) we have an $R_{e}$ of around 1.5 in the initial phase. Note that this is perfectly compatible with reports of alpha being 38–130% more infectious, since the acquired immunity was much higher during the alpha wave, which means that the virus needs to be significantly more infectious in order to be able to keep spreading.

### 4.3. Herd-Immunity under NPIs

Under Hypothesis 2, the decline in cases seen in January and again in May 2021, is due to depletion of susceptibles, given the fixed and rather mild NPIs (or “recommendations”, as they were officially known). In Figure 6, we also plot the model output without the vaccination scheme (dashed pink curve), indicating that the cases caused by the alpha-strain would have started to decline in April anyway, indicating that some form of herd-immunity had been reached. Another indication of this is the fact that there was no notable surge among unvaccinated school children under the age of 12 (prior to the omicron-wave), as discussed in Section 2.4 and Section 4.1. Note that antibodies against alpha were shown to provide good protection against delta, but less so against omicron [26,27].

In any case, there is always the possibility that another surge could have followed if people had returned to pre-pandemic mobility patterns (which there is no indication of in Figure 5), so the term herd-immunity may be misleading. Therefore, we will refer to herd-immunity “under NPIs”. By this we mean the point in the model at which cases naturally start to drop due to depletion of susceptibles, leading to a value of $R_{e}$ below 1 (despite human social patterns assumed constant over time). How to accurately compute the point at which this happens (against a certain strain) is rather subtle, and described in detail in Appendix A.9.

If Hypothesis 2 is correct, then Stockholm reached herd-immunity under NPIs against the Wuhan-strain in early December 2020, just before the closing of high schools, when the incidence curve leveled out. This is because the herd immunity threshold is reached at the peak of the wave, not after it has receded, (which is why a major outbreak usually ends with a final size substantially larger than the herd-immunity threshold). By the same logic, the herd-immunity threshold under NPIs for the alpha-strain was reached on April 9, 2021, when most people in the group 0–69 were still unvaccinated.

## 5. Discussion

The SARS-CoV-2 hit-rate in Sweden prior to omicron was around 30%, about equally distributed over three distinct waves, in stark contrast with state of the art SEIR-models which predict at least 60% hit rate in one massive wave. It seems generally believed that this can be explained by NPIs and voluntary public reduction in contact patterns, as well as ultimately the vaccination campaign. We investigate this hypothesis and find that, based on data from serological studies, the incidence-curve and Google-mobility data, the hypothesis seems unlikely, even without doing any modeling. We then run an age-stratified SEIR model taking vaccination and isolation of elderly into account, and found that only an unrealistic decrease in contact patterns could explain the cases seen in Stockholm during chosen time frame.

This challenges the interpretation of variable NPIs and human behavioral changes as the *main cause* for the rise and fall of epidemic waves, at least in locations where authorities have been either unwilling or incapable of enforcing strict NPIs, such as Sweden. Although NPIs and changes in social behavior clearly are important, we demonstrate that it is difficult to envision that they alone can explain the outbreaks seen in Stockholm County during the second and third waves.

However, this raises the question: is it always so that SEIR-models significantly overestimated the magnitude of spread, or is there something particular about SARS-CoV-2 that kept the incidence unexpectedly low?

Although it is clear that people in Sweden made major changes to their social interaction patterns, it seems to us that these changes were kept rather constant prior to the vaccination campaign. Some people worked from home and minimized unnecessary contacts, others organized private parties, but in both cases they did so irrespective of whether the incidence was high or low. However, under the assumption that human behavior was rather constant, standard mathematical models, such as SEIR, should, in theory, work well to predict the spread of COVID-19 in a densely populated area, such as Stockholm County. However, we have showed that it clearly does not.

Another interesting case is that of Brazil, which also reported surprisingly low levels of seroprevalence from most major cities during the first year of the pandemic (possibly with the exception of Manaus) despite applying limited NPIs. Similar comments can be made also about India, which could be explained if a level of pre-immunity was indeed present against the major strains of SARS-CoV-2 prior to omicron.

To mathematically investigate this possibility, we rely on a SEIR-model which is an extension of a state of the art model (developed for modeling COVID-19 by well known specialists in the field [5]), and find that the model, under the assumption that everybody is equally susceptible, significantly overestimates the disease spread (recall Figure 3). The only way that we are able to obtain a good model fit with real data is by including a pre-immunity assumption, and we find that this pre-immunity fraction needs to be substantial, in the range 55–65%, in order to obtain a good match with measured data.

### 5.1. What Could Have Caused Pre-Immunity?

We demonstrate in the adjacent publication [6] that a number of factors, such as people isolating, variable social activity patterns, as well as variations in susceptibility, are manifested as “artificial pre-immunity”. Hence, the hypothesis of pre-immunity does not necessarily imply that certain people had sterilizing protection against SARS-CoV-2. However, 65% is a large proportion, and it seems far-fetched to assume that people isolating to various degrees could amount to an average effect of 65% artificial pre-immunity. Additionally, the fact that seroprevalence among unvaccinated school children leveled out at around 30% until the omicron-wave, seems to indicate that a majority of these children had a high level of protection against contracting the initial strains of SARS-CoV-2 (i.e., Wuhan, alpha, and delta).

### 5.2. The Case of Omicron

Relying on the numbers from the Swedish Public Health Agency for the unvaccinated group aged 0–11 (see Supplementary Material), we infer that the amount of antibodies in this group increased by 42% (from 29.6 to 71.8) during the omicron surge December 2021 to March 2022. Since it is known that antibodies against prior variants of SARS-CoV-2 gave a very limited protection against infection by omicron, this tells us that somewhere between 42.2% and 71.8% were infected with omicron in this wave, and based on the authors personal observations it seems that the higher number is the more accurate figure. The fact that around 40–70% were infected with omicron in the course of 3 months also gives support to the hypothesis that actually SEIR-models *do work*, but some other, yet to be fully understood, factor was damping the initial waves of SARS-CoV-2. Since the actual amount of people who were infected with omicron is unknown, we refrain from fitting the measured omicron-wave with our model, but whether it is around 40 or 70% it is clear that this can be completed without heavily relying on any form of pre-immunity (especially in the latter case).

If it is the case that antibodies due to some cross reactive immunity protected a large part of the population during the first waves, then natural selection will favor variants that are able to circumvent this protection. Indeed, the omicron-mutations managed to circumvent the antibodies from prior variants. Therefore, it is natural to postulate that also the level of pre-immunity gradually deteriorates and eventually disappears. Thus, the fact that there was a massive omicron-wave is not in contradiction with Hypothesis 2.

### 5.3. Influenza-A Mediated Pre-Immunity

A number of factors, such as genetic, cross-reactive immunity and innate immunity, have been shown to provide variation in susceptibility [22,23,24]. Due to the findings presented in this article, we searched for the identity of a protective pre-immunity to SARS-CoV-2 and found that previous infections by influenza A H1N1 (Flu–strains) provides a specific antibody-mediated immunity preventing binding of the Spike protein to the Angiotensin Converting Enzyme 2 (ACE2) receptor. This antibody reactivity was found in 55–73% of people in Stockholm [7] and could, hence, be an important factor behind the 65% “artificial” pre-immunity identified in the present study. We also identified 12 different cross reactive T cell peptides that are shared between Influenza A H1N1 and SARS-CoV-2, which could provide HLA dependent T cell mediated protection against severe COVID-19 disease. We provide modeling data that Scandinavians carry HLA types that could mediate such protection in 71% of the population, while only in 40% of people among the world’s population [7].

We highlight that the fact that the hit-rate was rather low in Sweden, and that the death rates were lower in Sweden than in some other countries, fits well with this theory. In our study describing the identity of the Flu mediated protective pre- immunity, we make a case analysis with Stockholm and India to support this statement. Yet more compelling evidence that this theory could be true in reality comes from epidemiological observations demonstrating that Flu vaccinations have protected people from becoming infected with SARS-CoV-2 or becoming severely ill or dying from COVID-19 [28,29,30,31,32,33], with estimated protection rates of about 40–80%.

When omicron emerged, we were concerned that the mutations in the receptor binding domain of the Spike-protein (in region 477–505) would cause a loss of immunity mediated by previous infections, vaccinations, as well as the Flu mediated cross-protective immunity (localized to region 481–486) and create a highly infectious variant. As a consequence, we expected that many people would become infected with omicron, which is precisely what then happened. In this scenario, the observed data in Sweden and elsewhere is completely compatible with SEIR-models, and the fact that the outcome of the Swedish relaxed strategy was not a complete disaster could be attributed to luck, as this protective immunity was not known to the Swedish authorities. This possibility is important to be aware of when modeling infectious disease outbreaks and forming strategies to protect people in future pandemics.

## 6. Conclusions

We find that state of the art mathematical models, as well as standard formulas for the herd immunity threshold, are completely at odds with what happened in Sweden during the first year of the pandemic. The hypothesis that this is due to non-pharmacological interventions and/or voluntary updates in social patterns, is found unlikely to be true.

We are, thus, left with two possibilities: either state of the art models are inapt for modeling COVID-19, or a large proportion of the population had some a priori protection against contracting SARS-CoV-2. This is as far as the present work takes us, and we can only speculate over which answer is correct. Pre-immunity is one possible explanation for why there is such a large gap between model output and observed data, and, in this case, data are completely compatible with SEIR-models.

In summary, we argue that our findings favors the hypothesis that state of the art SEIR-models are good for modeling spread of SARS-CoV-2, but that a large fraction of the population indeed had some degree of protective pre-immunity, due to factors that remain to be understood in greater depth.

## Figures and Tables

**Table 1:** Summary of major updates to the NPIs in Sweden between September 2020 and May 2021.

1 (Oct-20)	Recommendations for elderly (70+) to self-isolate are removed and replaced by the same recommendations as for the general population.
2 (Nov-20)	Before 1 November the maximum amount of visitors to a public event was 50, which then was changed to 300, although a cap on maximum 50 participants in dancing events remained.
3 (Nov-20)	Prohibited public gatherings involving more than 8 people (shopping, restaurants, bars etc., were exempt from this rule).
4 (Dec-20)	Alcohol-sales only until 20.00, compared with 22.00 previously.
5 (Jan-21)	Facemask recommendation is issued for health care visits and rush-hour public transport.

**Figure A1:**
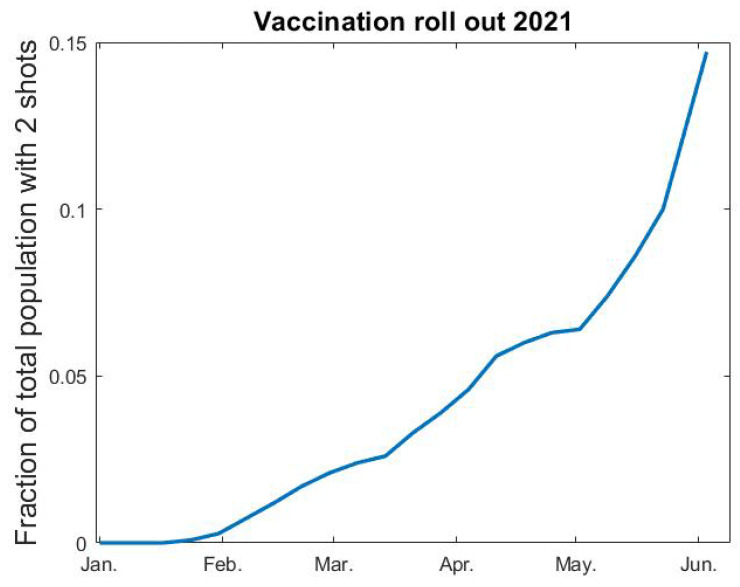
Fraction vaccinated *v* as function of time *t*.

## Data Availability

Codes and data are available on https://github.com/Marcus-Carlsson/Covid-modeling (accessed on 10 Augsut 2022) as well as in Supplementary Material.

## Supplementary Materials

The following supporting information can be downloaded at: https://www.mdpi.com/article/10.3390/v14081840/s1.

## Abbreviations

The following abbreviations are used in this manuscript:

NPI	Non-Pharmacological Intervention
ICU	Intensive Care Unit
HIT	Herd-Immunity Threshold

## Appendix A. Mathematical Modeling, Details

We build upon the age-stratified SEIR model developed by Britton et al. [5], which is described in greater detail in the Supplementary Material of [34]. In Appendix A.1–Appendix A.5, we explain how the model works in its most basic setup, enough to produce the red curve seen in Figure 3. In Appendix A.6, we then explain how to further update this in order to test Hypothesis 1, i.e., to produce the pink graph seen in Figure 3. How to model with pre-immunity and multiple strains is then explained in Appendix A.7, upon which the code for Figure 6 is built. The final two sections are mainly of mathematical interest and derive formulas for the herd-immunity threshold valid in the presence of (artificial) pre-immunity.

### Appendix A.1. Age-Stratified SEIR with Antibody Waning

Following Britton et al. [5], we divide the population into 6 age-groups and let s,e,i and *r* represent column-vectors including the fraction of the full population in respective age-group of the classes: Susceptible, Exposed, Infective, and Recovered. A contact matrix *A*, which we describe in detail in the coming section, determines the amount of contacts between the groups and a coefficient β the rate of transmission, so the equation corresponding to (1) becomes

(A1) $$\nu \left(t\right)=\beta {\mathrm{diag}}_{s(t)}Ai\left(t\right),$$

where ν is the rate of new cases in each respective group, and ${\mathrm{diag}}_{s(t)}$ denotes a diagonal matrix with the values in the vector s(t) in the diagonal.

The remaining functions in the model are e,i and **r**, which relate to **s** as follows:

(A2) $$\left\{\begin{array}{c} s^{\prime }\left(t\right)=-\nu \left(t\right)+\tau r\left(t\right)\\ e^{\prime }\left(t\right)=\nu \left(t\right)-\sigma e\left(t\right)\\ i^{\prime }\left(t\right)=\sigma e\left(t\right)-\gamma i\left(t\right)\\ r^{\prime }\left(t\right)=\gamma i\left(t\right)-\tau r\left(t\right) \end{array}\right.$$

A new feature is the term τr(t) which takes the waning of antibodies into account. Since we have decided to put half-life to 16 months, we set $\tau =\frac{\ln(2)}{16/12\times 365}$. Following [19], we set $\sigma =1/T_{incubation}=1/4.6$ and $\gamma =1/T_{infectious}=1/2.1$. The rationale behind these choices is explained in the Supplementary Material (Section 5) of [34].

### Appendix A.2. Calibration of the Contact Matrix

To model how the disease spreads within and between the different groups, we need some sort of measure of how often people in these groups interact. Wallinga et al [15] divided in 2006 the Dutch population into 6 groups aged 0–5, 6–12, 13–19, 20–39, 40–59, and 60+ and measured the amount of average weekly conversations held between group *j* and group *g*. To be precise, Wallinga et al. presents a “contact matrix” *M*, which is a 6×6 matrix whose j,k:th entry equals the amount of conversations between age group *j* and *k* on a weekly basis, divided bythe amount of people in group *k*. Hence $M_{j,k}$ is the average number of persons in group *j* that a person of group *k* has contacts with in a week (based on data from the Netherlands in 2006).

Several issues arise when modeling COVID-19 using this matrix: the age distribution in Sweden is clearly different than that of the Netherlands and, furthermore, even the Netherlands has a very different age distribution in 2020 than in 2006. For example, in [15], it is stated that the age group 60+ is about 17%, whereas the age group 70+ in Sweden 2020 make up 14%, due to an aging population. We, thus, need to decide on how to update *M* given a new age distribution *w*.

The elements of *M* represent “the average number of persons in group *j* that a person of group *k* has contacts with”, in the Netherlands in 2006. If we postulate that these numbers are roughly the same in Stockholm County 2020, then $M_{j,k}w_{k}N$ is roughly the amount of conversations between groups *j* and *k*, whereas by reciprocity $M_{k,j}w_{j}N$ represents the same number, but these two numbers will no longer be the same. (Here *N* denotes the total population N=2,400,000). To remedy this issue, we suggest to define $M_{j,k}^{new}$ by taking the average and then divide by the amount of people in group *k*, i.e., $w_{k}N$. This leads to the formula

(A3) $$M_{j,k}^{new}=\frac{M_{k,j}w_{j}+M_{j,k}w_{k}}{2w_{k}}.$$

For modeling of COVID-19 in Sweden, it is clearly better to have 70 as a delimiter for the last age group, since special recommendations applied to this group which in particular was encouraged to minimize all social contacts during a large part of the pandemic. Therefore, the *w* we used for the modeling in this paper was chosen to correspond with the distribution in the age-groups 0–5, 6–12, 13–19, 20–39, 40–69, and 70+.

Based on $M^{new}$, we can now introduce the contact matrix *A* by the formula

$$A_{j,k}=\frac{M_{j,k}^{new}}{7w_{j}}$$

which, thus, is a symmetric matrix (since $M_{j,k}^{new}w_{k}=M_{k,j}^{new}w_{j}$), where the factor 7 is convenient, since it allows us to work with daily contacts instead of weekly. This choice is discussed in greater depth in the Supplementary Material of [34].

The above matrix is likely a good contact matrix for the Swedish population in the absence of NPIs. However, one also needs to take into account that the group 70+ were recommended to self-isolate during large parts of the pandemic, and, hence, it is likely that they reduced their contacts much more than the other age groups. We incorporate this by multiplying all numbers in the last row and column of *A* by a factor ξ, which we set to ξ=1/4 for reasons to be explained shortly. Our model, thus, assumes that the 70+ group reduced social contacts 4 times more than other age-groups.

In order to test whether our contact matrix *A* actually represents social interaction patterns in Sweden during the pandemic, we run our model and compare the fraction of infected in each subgroup with actual data. Relying on data from the Public Health Agency data from February 2 2021 (before the effect of vaccinations became relevant), we inferred that 24% of the total cumulative COVID-19 cases were from the age group 0–19, 31% in the age group 20–39, 37% in the age group 40–69, and 8% of the cases were in the group 70+. Upon running our model, we found that the reduction factor ξ=1/4 gives values 23%, 32%, 37%, and 8% on the same date. This is sufficiently close to the observed numbers mentioned above, which, thus, gives support for using the contact matrix with the modifications described above.

### Appendix A.3. Incorporating Vaccinations

We rely on data from the Public Health Agency [35] for percentages of the population vaccinated each week (see Figure A1 below). After interpolation, we let v(t) denote fraction of vaccinated as a function of time. Since vaccine efficacy varies between different vaccines and different age groups, it is not entirely straightforward how to include vaccines in a model where immunity is a binary value. The AstraZeneca vaccine was given to elderly people and to health care personnel in the beginning of 2021, whereafter the mRNA vaccines from Pfizer and Moderna became the major vaccines given to all age groups in Sweden, but the second dose of these vaccines were given after June 2021 and, hence, we do not include this in our modeling. The AstraZeneca vaccine was estimated to have a 70% protective effect to the original SARS-CoV-2 strain, and a reduced protection rate is expected against the alpha variant. In our model, the highest age-group 70+ consist of 15% (this is group number 6), which coincides with the amount of complete vaccinations early June 2021 (see Figure A1). With this in mind, we postulate that 70% of the vaccinated people become immune, and that the vaccination only affects group 6. In the medRxiv preprint of this paper [36] we instead assumed a 90% efficacy after the first shot, and, therefore, also included vaccinations in group 5 in our modeling. We updated this since the way we do it here seems more realistic and also much simpler to describe and code. This only has a marginal effect on the curve and does not in any way alter the conclusions of the paper.

In practice, we thus add an “artificial” flow from the 6th group $s_{6}$ of *s* to the corresponding group in *r*, which hence represents both recovered and vaccinated. Another thing to keep in mind is that the vaccination scheme did not separate between people who had had COVID-19 and those who had not. Therefore, some people who were vaccinated were already in the corresponding age-group of the **r**-compartment. Since $s_{6}\left(t\right)/w_{6}$ is the fraction of elderly that are still susceptible to the virus at time *t*, and $v^{\prime }\left(t\right)$ is the vaccination rate, we postulate that $0.7v^{\prime }\left(t\right)s_{6}\left(t\right)/w_{6}$ is the rate at which the vaccination roll-out provides protective antibodies to individuals in group 6. We, therefore, subtract the term $0.7v^{\prime }\left(t\right)s_{6}\left(t\right)/w_{6}$ from the last row of the equations for $s^{\prime }$, and simultaneously add it to the last row of the $r^{\prime }$ equations.

### Appendix A.4. Approximative Solutions to (uid27) and (uid28)

Since *s* is changing very slowly, we can linearize the Equation (A1) around a given time value $t_{0}$. The equations for the disease compartments **e** and **i** when $t\approx t_{0}$ then become:

(A4) $$\frac{d}{dt}\left[\begin{array}{c} e\\ i \end{array}\right]=\left[\begin{array}{cc} \sigma I & \beta {\mathrm{diag}}_{s(t_{0})}A\\ -\sigma I & \gamma I \end{array}\right]\left[\begin{array}{c} e\\ i \end{array}\right],$$

written using standard matrix-vector notation, where *I* denotes the identity matrix. For simplicity we shall denote the above matrix by $M_{t_{0}}$. As is well known by standard theory of Ordinary Differential Equations, the dominant solution to the above equation system is given by the eigenvector with the largest eigenvalue, given that this is real and unique. We now prove that this is the case.

**Theorem** **A1.**
*The eigenvalues $\alpha_{1},\ldots ,\alpha_{6}$ of $\beta {\mathrm{diag}}_{s(t_{0})}A$ are real and the largest eigenvalue is positive and has multiplicity 1. The eigenvalues of $M_{t_{0}}$ are given by*

(A5)
$$-\frac{\sigma +\gamma }{2}\pm \sqrt{{\left(\frac{\sigma -\gamma }{2}\right)}^{2}+\sigma \alpha_{k}},\;k=1,\ldots ,6.$$

*The eigenvalue with the largest real part, i.e. $\lambda_{1}=-\frac{\sigma +\gamma }{2}+\sqrt{{\left(\frac{\sigma -\gamma }{2}\right)}^{2}+\sigma \alpha_{1}}$, has multiplicity one and the corresponding eigenvector can be chosen to have positive entries.*

**Proof.** The eigenvectors $x_{k}$ of $\beta {\mathrm{diag}}_{s(t_{0})}A$ also solve the generalized eigenvalue problem

$$\alpha_{k}{\left({\mathrm{diag}}_{s(t_{0})}\right)}^{-1}x_{k}=\beta Ax_{k}.$$

Since *A* is symmetric, it follows by standard theory of such equation systems that there exists a basis of 6 real eigenvectors with corresponding real eigenvalues, see, e.g., ref. [37]. Moreover, the largest eigenvalue is positive, has multiplicity one and $|\alpha_{k}|<\alpha_{1}$ for all k>2, by the Perron–Frobenius theorem, which also states that the corresponding eigenvector $x_{1}$ can be chosen to have positive entries. We now show that each such pair $\left(\alpha_{k},x_{k}\right)$ gives rise to two eigenvalues/eigenvectors of $M_{t_{0}}$. Indeed, if we consider a vector of twice the size given by $\left[\begin{array}{c} ax_{k}\\ bx_{k} \end{array}\right]$, it follows that this is an eigenvector to $M_{t_{0}}$ if, and only if, $\left[\begin{array}{c} a\\ b \end{array}\right]$ is an eigenvector of

(A6) $$\left[\begin{array}{cc} -\sigma & \alpha_{k}\\ \sigma & -\gamma \end{array}\right].$$

The corresponding eigenvalue of $M_{t_{0}}$ is then the eigenvalue of the above 2 × 2-matrix. It follows by basic matrix theory that this matrix has the eigenvalues stated in (A5). Since $\alpha_{1}$ has multiplicity one and is positive, it follows by (A5) that $\lambda_{1}$ also has multiplicity one. Let $\left[\begin{array}{c} a_{1}x_{1}\\ b_{1}x_{1} \end{array}\right]$ be the corresponding eigenvector. It remains to show that also $a_{1}$ and $b_{1}$ can be chosen positive. By some basic computations that we omit, we can pick $a_{1}=\lambda_{1}+\gamma$ and $b_{1}=\sigma$, and it is easy to see that

$$\lambda_{1}+\gamma =-\frac{\sigma -\gamma }{2}+\sqrt{{\left(\frac{\sigma -\gamma }{2}\right)}^{2}+\sigma \alpha_{1}}>0.$$

□

To conclude this section, we remark that it follows by standard theory of Ordinary Differential Equations that the solution to (A4) will be dominated by a multiple of $e^{\lambda_{1}t}\left[\begin{array}{c} a_{1}x_{1}\\ b_{1}x_{1} \end{array}\right]$ for $t>>t_{0}$, independent of the initial distributions at time $t_{0}$.

### Appendix A.5. Initial Conditions

At the start of our modeling, on 1 September 2020, we know that 10.2% in Stockholm were already recovered and that the rate of new infections was around 80 per day. However, we do not know the distribution of the 10.2% among the different age groups, which we need to pick an initial value for *s*, and it is also not clear how to chose initial values of *e* and *i* to match 80 cases per day. In this section, we describe how this is performed. (Since this becomes rather technical, we want to stress that the way the initial conditions are chosen have no major bearing on the model curves or conclusions of the paper. In the medRxiv preprint version of this paper [36], we chose initial conditions by simply running the model, and the corresponding graphs are almost identical).

At the onset of the pandemic in February 2020, we have s=w (at least in the absence of pre-immunity, which we will discuss how to include in a later section). By the results in the previous section, it is therefore reasonable to assume that the distribution of cases in the first wave roughly was distributed as $x_{1}^{Feb}$, where $x_{1}^{Feb}$ is the eigenvector corresponding to the largest eigenvalue of $\beta {\mathrm{diag}}_{w}A$. Hence, we set the initial value for **r** to $\psi \cdot x_{1}^{Feb}$, where ψ=0.102 and we choose $x_{1}^{Feb}$ so that its entries sum up to 1. Since the vectors s,e,i, and **r** must sum up to **w**, this means that, when cases start to accumulate again in September of 2020, we should have $s\approx w-\psi \cdot x_{1}^{Feb}$ (since the amount in **e** and **i** are negligible). Consequently, again following the logic of the previous section, the distribution of cases in $\left[\begin{array}{c} e\\ i \end{array}\right]$, in the beginning of September 2020, should be a constant multiple of $\left[\begin{array}{c} a_{1}x_{1}^{Sep}\\ b_{1}x_{1}^{Sep} \end{array}\right]$ where now $x_{1}^{Sep}$ is the eigenvector of $\beta {\mathrm{diag}}_{w-\psi \cdot x_{1}^{Feb}}A$ with largest eigenvalue $\alpha_{1}$, and $a_{1}$, $b_{1}$ are corresponding positive numbers, just as in the proof of Theorem A1. Since $s\left(0\right)\approx w-\psi \cdot x_{1}^{Feb}$, the constant multiple *c* can now be found by setting $i\left(0\right)=cb_{1}y_{1}$ and choosing *c* so that the vector $\beta {\mathrm{diag}}_{w-\psi \cdot x_{1}}Ai\left(0\right)$ (representing the fraction of new cases on day 0, recall (A1)) sums up to 80/N (where N=2,400,000; the population in Stockholm County). Finally, we pick the initial condition for *s* by the requirement that the vectors s,e,i, and **r** must sum up to **w**. (Doing this with the actual numbers gives that there are about 2.4 more people in the exposed group than the infected group, which is almost the same as what one obtains by running the model with one initial case and stopping when reaching around 80, as was done in [36]).

### Appendix A.6. Modeling with Variable Human Contact Patterns

For testing Hypothesis 1 we also need to update (A1) so that the transmission rate β is allowed to vary over time. We replace the constant β with the product of two functions β(t) and (1−f(t)/100), where the former describes the variation in transmission rate due to the introduction of alpha and the latter only model changes in behavior/NPI. Concretely, we replace (A1) with

(A7) $$\nu \left(t\right)=\beta \left(t\right)\left(1-f\left(t\right)/100\right){\mathrm{diag}}_{s(t)}Ai\left(t\right),$$

The function β(t) will be constant until the arrival of alpha in early January, by which time it will start to grow and reach a new level once alpha took over, in early April. Since we know the fraction of alpha over time we can form β(t) easily by setting

$$\beta \left(t\right)=\beta_{Wuhan}\frac{Cases_{Wuhan}\left(t\right)}{Cases_{Tot}\left(t\right)}+\beta_{alpha}\frac{Cases_{alpha}\left(t\right)}{Cases_{Tot}\left(t\right)}$$

where $Cases_{Wuhan},\;Cases_{alpha}$ and $Cases_{Total}$ represent the red, yellow, and blue curves in Figure 2 (right), respectively. The constants $\beta_{Wuhan}$ and $\beta_{alpha}$, represent the transmission rate of the Wuhan strain and alpha strain, respectively. In Figure 4, we have used $\beta_{alpha}=1.38\beta_{Wuhan}$ (upper black), $\beta_{alpha}=1.5\beta_{Wuhan}$ (red), and $\beta_{alpha}=2.3\beta_{Wuhan}$ (lower black), which is based on estimates from [25] about how much more contagious the alpha strain is. Finally, we pick a value of $\beta_{Wuhan}$ so that we obtain a good initial fit with real data upon setting f(t)=0 for *t* in September.

Now, given a fixed choice of *f* we can run our model and compare its outcome with real data. The concrete estimate of *f* seen in red in Figure 4 (left) was obtained by first running the model “backwards” to compute an *f* that gave an identical fit (using $\beta_{alpha}=1.5\beta_{Wuhan}$). This *f* was then averaged over a 30-day window and extrapolated linearly on the edges. Of course, exactly how *f* is obtained is not so interesting as long as it gives a good fit with real data, which indeed is the case, as seen in Figure 3 (pink).

### Appendix A.7. Incorporating Artificial Pre-Immunity and Multiple Strains

To deal with pre-immunity and multiple strains, the basic setup (A1) and (A2) needs to be modified slightly, as we now explain. Adding pre-immunity is most easily done by initializing **s** by s(0)=(1−θ)w where θ is the fraction of pre-immunity and *w* the vector with the fraction of the population in the respective age-group (since we have no reason to assume that the pre-immunity is not evenly distributed among different age-groups). However, as explained in [6], the pre-immunity θ (which we have estimated to 0.65 for Stockholm and the Wuhan-strain) is most likely an over-simplification resulting from the phenomenon that susceptibility to the virus is variable between individuals. Upon introducing a variant of concern, we obtain a new value of β but potentially also a new value for θ, since the new strain may be better at infecting some individuals that had a good protection against the previous strain (see [6], Section 4.2, for a fuller discussion of this topic). Since we can not suddenly change the initial value in the model, the above way of introducing pre-immunity is unsuitable for dealing with multiple strains. Therefore, we instead incorporate the pre-immunity θ by replacing the term (A1) by

(A8) $$\nu \left(t\right)=\beta {\mathrm{diag}}_{s(t)-\theta w}Ai\left(t\right).$$

As long as the model is only dealing with one strain, this is equivalent to using s(1)=(1−θ)w as initial condition, but this approach allows for multiple θ’s related to various strains.

To include the alpha strain, we introduce new parameters $\beta_{alpha}$ and $\theta_{alpha}$, as well as functions, $\nu_{alpha}$, $e_{alpha}$, and $i_{alpha}$, which describe the incidence, as well as the fraction of exposed and infected in the population that contracted the alpha strain. As before we set

(A9) $$\nu_{alpha}\left(t\right)=\beta_{alpha}{\mathrm{diag}}_{s\left(t\right)-\theta_{alpha}w}Ai_{alpha}\left(t\right),$$

and obtain an equation system where both the Wuhan strain and the alpha strain exist in parallel as follows:

$$\left\{\begin{array}{c} s^{\prime }\left(t\right)=-\nu \left(t\right)-\nu_{alpha}\left(t\right)+\tau r\left(t\right)\\ e^{\prime }\left(t\right)=\nu \left(t\right)-\sigma e\left(t\right)\\ e_{alpha}^{\prime }\left(t\right)=\nu_{alpha}\left(t\right)-\sigma e_{alpha}\left(t\right)\\ i^{\prime }\left(t\right)=\sigma e\left(t\right)-\gamma i\left(t\right)\\ i_{alpha}^{\prime }\left(t\right)=\sigma e_{alpha}\left(t\right)-\gamma i_{alpha}\left(t\right)\\ r^{\prime }\left(t\right)=\gamma i\left(t\right)+\gamma i_{alpha}\left(t\right)-\tau r\left(t\right) \end{array}\right.$$

This is the main update needed in order to produce the curves in Figure 6. For simplicity, we have not included the terms related to the vaccination roll out in the above equation system, which was explained how to do in Appendix A.3 above. We have also omitted to discuss the rather straightforward modifications to Appendix A.5 that needs to be made to include 10.2% acquired immunity in the initial conditions. We refer to the code Fig6.m, found on https://github.com/Marcus-Carlsson/Covid-modeling (accessed on 10 Augsut 2022).

### Appendix A.8. Computing R 0

In the coming section we discuss how to estimate when herd-immunity is reached, given the model (A1) and (A2) with pre-immunity, i.e., we do not consider the model involving multiple strains, and we include pre-immunity as explained in (A8). Recall that the classical formula states that the herd-immunity threshold is given by $1-1/R_{0}$. To update this we first need to work out how $R_{0}$ depends on β, θ, *A*, following Section 5.2 [14], which is done in this section.

The equations for $e^{\prime }$ and $i^{\prime }$ using (A8) become

$$\left[\begin{array}{c} e^{\prime }\left(t\right)\\ i^{\prime }\left(t\right) \end{array}\right]=\left[\begin{array}{c} \beta {\mathrm{diag}}_{s(t)-\theta w}Ai\left(t\right)-\sigma e\left(t\right)\\ \sigma e(t)-\gamma i(t) \end{array}\right]=\left[\begin{array}{cc} -\sigma I & \beta {\mathrm{diag}}_{s(t)-\theta w}A\\ \sigma I & -\gamma I \end{array}\right]\left[\begin{array}{c} e(t)\\ i(t) \end{array}\right]$$

(where the term −θw is added since we now model using (A8) instead of (A1) to compute daily new cases). As explained in Appendix A.4 this system can be approximated by a linear ODE, at any time $t=t_{0}$, by simply freezing the value of s(t), giving rise to

(A10) $$\left[\begin{array}{c} e^{\prime }\left(t\right)\\ i^{\prime }\left(t\right) \end{array}\right]=\left[\begin{array}{cc} -\sigma I & \beta {\mathrm{diag}}_{s(t_{0})-\theta w}A\\ \sigma I & -\gamma I \end{array}\right]\left[\begin{array}{c} e(t)\\ i(t) \end{array}\right]:=M_{t_{0}}\left[\begin{array}{c} e(t)\\ i(t) \end{array}\right]$$

valid for $t\approx t_{0}$, where the matrix $M_{t_{0}}$ is defined by the above line. Since $s(t_{0})=w$ in the beginning of the pandemic we set $F=\left(\begin{array}{cc} 0 & \beta {\mathrm{diag}}_{(1-\theta )w}A\\ 0 & 0 \end{array}\right)$ and $V=\left(\begin{array}{cc} \sigma I & 0\\ -\sigma I & \gamma I \end{array}\right)$, so that F−V equals $M_{t_{0}}$, (using the notation in [14], in particular Example 1, Section 5.2). The next generation matrix, denoted $K_{L}$, is now defined as

$$K_{L}=FV^{-1}=\left(\begin{array}{cc} \frac{1}{\gamma }{\mathrm{diag}}_{(1-\theta )w}\beta A & \frac{1}{\gamma }{\mathrm{diag}}_{(1-\theta )w}\beta A\\ 0 & 0 \end{array}\right).$$

The $R_{0}$ value is then defined as the modulus of the largest eigenvalue of this matrix, also known as the spectral radius $\rho (K_{L})$. Using the well-known formula $\rho \left(K_{L}\right)=\lim_{n\to \infty }(∥K_{L}^{n}{∥)}^{1/n},$ it is easy to see that

$$\rho \left(K_{L}\right)=\rho \left(\frac{1}{\gamma }{\mathrm{diag}}_{(1-\theta )w}\beta A\right)=\left(1-\theta \right)\beta T_{infectious}\rho \left({\mathrm{diag}}_{w}A\right)$$

and, hence, it follows that

(A11) $$R_{0}=\left(1-\theta \right)\beta T_{infectious}\rho \left({\mathrm{diag}}_{w}A\right).$$

### Appendix A.9. The Herd Immunity Threshold

In this section, we discuss how to estimate when herd-immunity is reached. We do not consider the model involving multiple strains because both times herd-immunity seems to have been reached in Stockholm we had only one dominant strain. Even under this simplification, it is not entirely obvious of how to define the herd-immunity threshold, due to the fact that we work with multi-compartment models (various age-groups).

Intuitively, herd-immunity is reached when the amount of susceptibles are few enough that the amount of people who become sick, i.e., the total in *e* and *i*, start to recede. This is equivalent to demand that if we have no spread and a group of infectives are added to the system, the amount of secondary cases will be a decreasing function. The issue of at what level this happens becomes delicate since this does not only depend on the total amount of remaining susceptibles, but also on their distribution between the various groups. Classical estimates of HIT, (see [14] Ch. 5), assume a uniform distribution of immunity, whereas during a real outbreak immunity will be higher in groups that are more active in spreading the virus. In practice, this means that the intuitive definition will be fulfilled before the classical estimate is fulfilled.

To take a concrete example, the mathematical (homogenous) HIT is reached on day 191 of our modeling, i.e., 10th of March, but the amount of individuals in all subgroups of both *e* and *i* start to decrease on day 94, i.e., the second of December, just before the first update in the NPIs that could have had a limiting effect on the spread (closure of high-schools, see Figure 2). We now describe mathematically how to compute the HIT in two different ways, starting with the more intuitive one corresponding with day 94 in the earlier example.

To locally analyze the progression, we linearize around a given time point $t_{0}$, as in (A10). As explained in Appendix A.4, the solution to such an equation system consists of linear combinations of exponential functions whose exponents are the eigenvalues of the corresponding matrix $M_{t_{0}}$. Hence, the solutions will be decreasing independent of initial data whenever all eigenvalues have negative real part. The following theorem indicates precisely when this happens.

**Theorem** **A2.**
*Assume that A is a symmetric positive matrix. Given a time $t_{0}$ and a distribution of susceptibles $s(t_{0})$, we have reached herd immunity if, and only if,*

(A12)
$$\rho \left({\mathrm{diag}}_{s(t_{0})-\theta w}A\right)<1/\beta T_{infectious}.$$

Note that the contact matrix *A* indeed should be symmetric and positive, as shown in Appendix A.2. Hence, the theorem basically states that the point at which all compartments in the disease states *e* and *i* start to decrease, equals the first value of $t_{0}$ for which (A12) is fulfilled.

**Proof.** By the arguments before the statement, we need to show that the eigenvalues of $M_{t_{0}}$ all have negative real part if, and only if, (A12) holds. By Theorem A1 we thus need to show that $\lambda_{1}<0$ if and only if (A12) holds, where $\lambda_{1}=-\frac{\sigma +\gamma }{2}+\sqrt{{\left(\frac{\sigma -\gamma }{2}\right)}^{2}+\sigma \alpha_{1}}$ and $\alpha_{1}$ is the largest eigenvalue of $\beta {\mathrm{diag}}_{s(t_{0})-\theta w}A$. Rewriting $\lambda_{1}$ as

$$\lambda_{1}=-\frac{\sigma +\gamma }{2}\pm \sqrt{{\left(\frac{\sigma +\gamma }{2}\right)}^{2}+\sigma \left(\alpha_{k}-\gamma \right)}$$

we see that $\lambda_{1}<0$ if, and only if, $\alpha_{1}<\gamma$. Since $\gamma =1/T_{infectious}$ and $\alpha_{1}$ equals the spectral radius $\beta {\mathrm{diag}}_{s(t_{0})-\theta w}A$ (by the Perron–Frobenius theorem), this is equivalent to (A12), and the proof is complete. □

The above theorem can easily be used in practice given a concrete model fit of real data, as in Figure 6. Then, all quantities in (A12) are known and the first date that it is fulfilled is easily computed. This is how we derived that herd immunity against the Wuhan-strain was reached on December 2, 2020, and similarly we find that herd-immunity against alpha occurred on April 9, 2021.

In order to derive the classical herd-immunity threshold we now assume that immunity is homogeneously distributed over the groups. Let ζ be the fraction of immune needed to have herd-immunity. To derive a formula for ζ, under the above assumption, we simply replace $s(t_{0})$ in (A12) with (1−ζ)w. This leads to the equation

$$\rho \left({\mathrm{diag}}_{(1-\zeta -\theta )w}A\right)<1/\beta T_{infectious},$$

which, upon recalling (A11), can be recast in the following more attractive form

(A13) $$\zeta =\left(1-\theta \right)\left(1-\frac{1}{R_{0}}\right).$$

Relying on this formula, one sees that the effect of artificial pre-immunity θ is to reduce the herd-immunity threshold by the corresponding fraction, which is why this threshold in reality is much lower than traditional models predict (if the pre-immunity hypothesis is correct). For example, given the parameters used in this paper for the second wave of Stockholm, the herd-immunity threshold as given by (A13) is around 20%, whereas (A12) is satisfied when the total immunity level is merely 14%. This happens on day 94 in our model (i.e. on December 2nd, when all disease compartments de facto start to shrink), whereas a total of 20% of immunity is reached on day 191 of our model, i.e. long after the second wave was over. In summary, we argue that (A12) is the more realistic definition of when the herd-immunity threshold is reached in a concrete modeling example, whereas (A13) is a good first order approximation (upper bound) to the actual figure.
