# Supplementary material for: COVID-19 Modeling Outcome versus Reality in Sweden
Source: Viruses. 2022 Aug 22;14(8):1840. doi: 10.3390/v14081840 (PMC9415753; doi:10.3390/v14081840)
Supplement: Supplementary file 1 [file viruses-14-01840-s001.zip › viruses-1797662-supplementary.pdf]

Covid-19 modeling outcome versus reality in Sweden

This document provides the details of where to find the data upon which the code for “Covid-19 modeling outcome versus reality in Sweden” is based. All modeling is done using MatLab and related code as well as necessary time series is found in https://github.com/Marcus-Carlsson/Covid-modeling.

The raw data of cases comes from the Swedish Public Health Agency’s downloadble excel-document Folkhalsomyndigheten_Covid19.xlsx. A version of this file, downloaded on 02-Aug-2022, is available in the supplementary material. Permission to reproduce this file has been given.

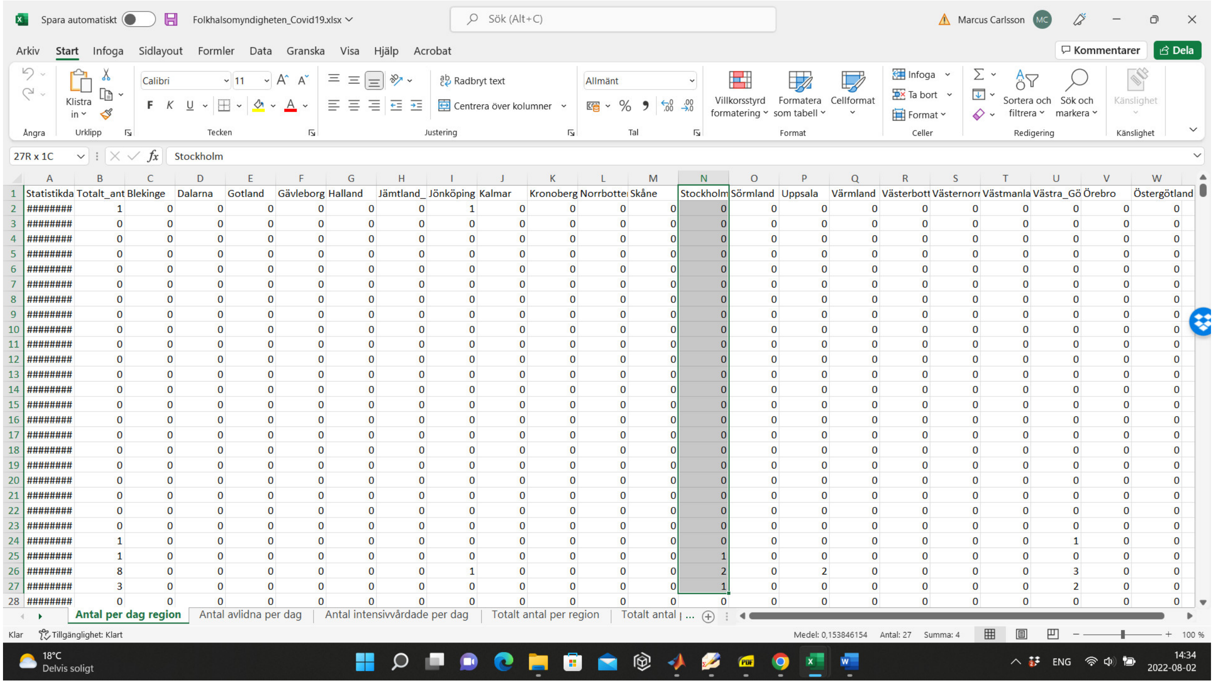

This column is stored in the file stockholm_data_raw.mat. The first entry is from the 4th of Febuary 2020 and the last is from 27th of July 2022. Due to an error in May 2021, the data was erroneously stored in a number of days, which is corrected using the file sminet_failure_correction.m, producing the file stockholm_data_corrected.mat. Data is then averaged over a 7-day window in average_stockholm_data.m, creating stockholm_data_weekly_average.mat as well as stockholm_data_weekly_average_sept2020_to_may_2021.mat. The latter contains the data between 1st of september 2020 and 3rd of June 2021, which is the timeframe we focus on in the main article.

To estimate the amount of cases due to the original Wuhan strain, we need data on Variants of Concern, which at the time of writing was available at https://www.folkhalsomyndigheten.se/smittskydd-beredskap/utbrott/aktuella-utbrott/covid-19/statistik-och-analyser/sars-cov-2-virusvarianter-av-sarskild-betydelse/tidigare-statistik/vecka-13/

Screenshots are provided below for convenience. The first screenshot contains data on alpha or B.1.1.7. Note that the column of interest is Stockholm containing the values 27, 41, 53, 66, 79, 84, 88 and 92. In Sweden, the time is often counted in weeks, starting from the first week of the year. The

data point 27 is for weeks 5 and 6, which equals the 1-14th of February (2021). The following data points are for the weeks 7 up to 13. Since data is missing outside of this interval we interpolate from 0% in the first day of week 3 to 23% in week 5 and 31 in week 6, making the average 27 as reported above. This is a bit ad hoc, but we do this to avoid confusing irregularities in the plots. Probably the first cases of alpha predate week 3, but if the interpolation is done differently it has marginal effect on the graphs and no bearing on any of the conclusions in the paper. Similarly we interpolate linearly from the last measurement down to 0 in early June 2021.

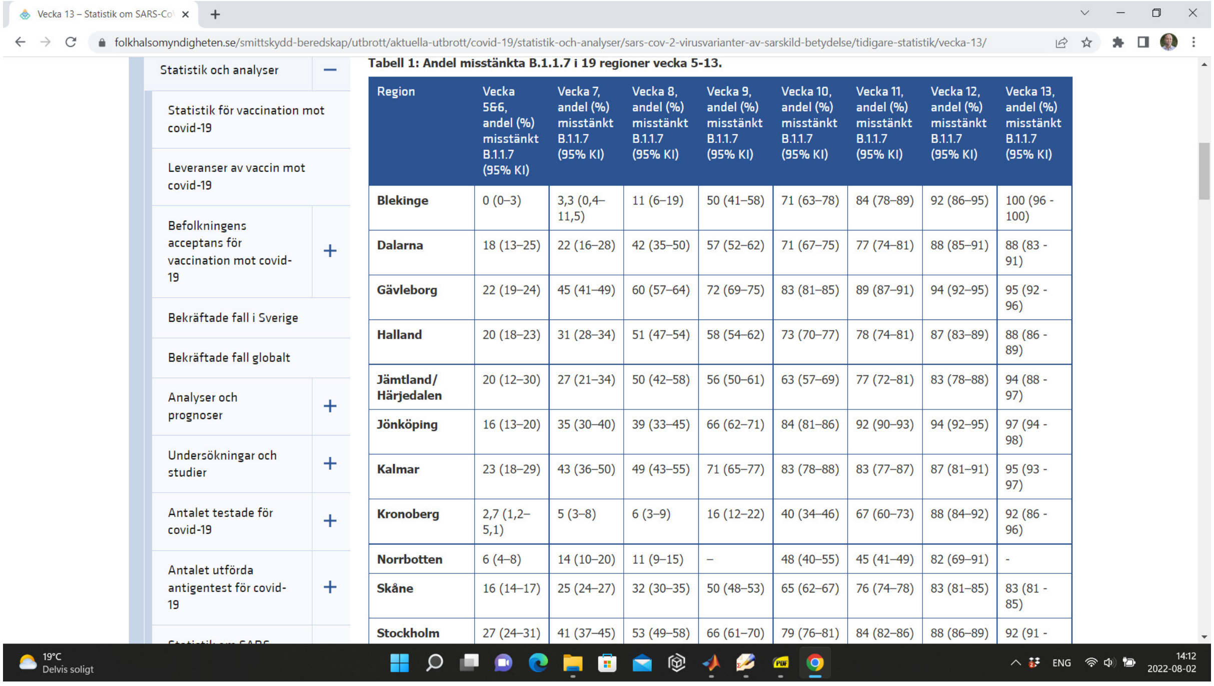

Tabell 1: Andel misstänkta B.1.1.7 i 19 regioner vecka 5-13.

Region	Vecka 5&6, andel (%) misstänkt B.1.1.7 (95% KI)	Vecka 7, andel (%) misstänkt B.1.1.7 (95% KI)	Vecka 8, andel (%) misstänkt B.1.1.7 (95% KI)	Vecka 9, andel (%) misstänkt B.1.1.7 (95% KI)	Vecka 10, andel (%) misstänkt B.1.1.7 (95% KI)	Vecka 11, andel (%) misstänkt B.1.1.7 (95% KI)	Vecka 12, andel (%) misstänkt B.1.1.7 (95% KI)	Vecka 13, andel (%) misstänkt B.1.1.7 (95% KI)
**Blekinge**	0 (0–3)	3,3 (0,4–11,5)	11 (6–19)	50 (41–58)	71 (63–78)	84 (78–89)	92 (86–95)	100 (96 - 100)
**Dalarna**	18 (13–25)	22 (16–28)	42 (35–50)	57 (52–62)	71 (67–75)	77 (74–81)	88 (85–91)	88 (83 - 91)
**Gävleborg**	22 (19–24)	45 (41–49)	60 (57–64)	72 (69–75)	83 (81–85)	89 (87–91)	94 (92–95)	95 (92 - 96)
**Halland**	20 (18–23)	31 (28–34)	51 (47–54)	58 (54–62)	73 (70–77)	78 (74–81)	87 (83–89)	88 (86 - 89)
**Jämtland/ Härjedalen**	20 (12–30)	27 (21–34)	50 (42–58)	56 (50–61)	63 (57–69)	77 (72–81)	83 (78–88)	94 (88 - 97)
**Jönköping**	16 (13–20)	35 (30–40)	39 (33–45)	66 (62–71)	84 (81–86)	92 (90–93)	94 (92–95)	97 (94 - 98)
**Kalmar**	23 (18–29)	43 (36–50)	49 (43–55)	71 (65–77)	83 (78–88)	83 (77–87)	87 (81–91)	95 (93 - 97)
**Kronoberg**	2,7 (1,2–5,1)	5 (3–8)	6 (3–9)	16 (12–22)	40 (34–46)	67 (60–73)	88 (84–92)	92 (86 - 96)
**Norrbotten**	6 (4–8)	14 (10–20)	11 (9–15)	–	48 (40–55)	45 (41–49)	82 (69–91)	-
**Skåne**	16 (14–17)	25 (24–27)	32 (30–35)	50 (48–53)	65 (62–67)	76 (74–78)	83 (81–85)	83 (81 - 85)
**Stockholm**	27 (24–31)	41 (37–45)	53 (49–58)	66 (61–70)	79 (76–81)	84 (82–86)	88 (86–89)	92 (91 -

The following screenshot concerns variants of concern other than B.1.1.7.

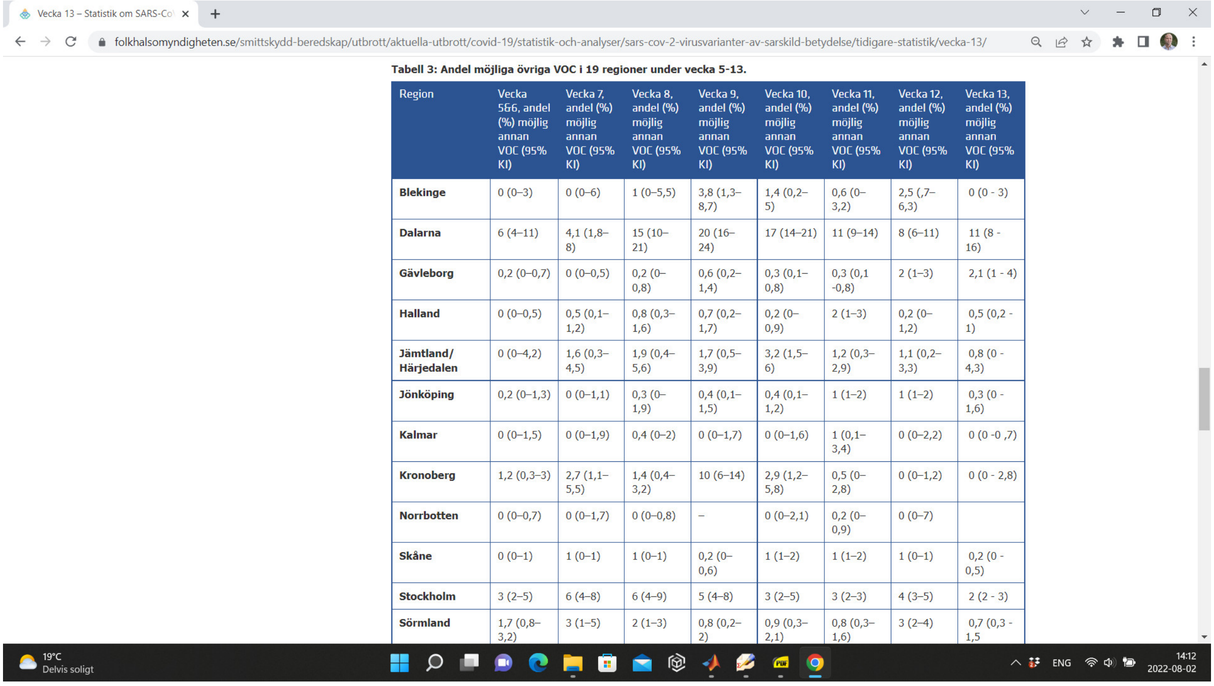

Tabell 3: Andel möjliga övriga VOC i 19 regioner under vecka 5-13.

Region	Vecka 5&6, andel (%) möjlig annan VOC (95% KI)	Vecka 7, andel (%) möjlig annan VOC (95% KI)	Vecka 8, andel (%) möjlig annan VOC (95% KI)	Vecka 9, andel (%) möjlig annan VOC (95% KI)	Vecka 10, andel (%) möjlig annan VOC (95% KI)	Vecka 11, andel (%) möjlig annan VOC (95% KI)	Vecka 12, andel (%) möjlig annan VOC (95% KI)	Vecka 13, andel (%) möjlig annan VOC (95% KI)
**Blekinge**	0 (0–3)	0 (0–6)	1 (0–5,5)	3,8 (1,3–8,7)	1,4 (0,2–5)	0,6 (0–3,2)	2,5 (,7–6,3)	0 (0 - 3)
**Dalarna**	6 (4–11)	4,1 (1,8–8)	15 (10–21)	20 (16–24)	17 (14–21)	11 (9–14)	8 (6–11)	11 (8 - 16)
**Gävleborg**	0,2 (0–0,7)	0 (0–0,5)	0,2 (0–0,8)	0,6 (0,2–1,4)	0,3 (0,1–0,8)	0,3 (0,1 -0,8)	2 (1–3)	2,1 (1 - 4)
**Halland**	0 (0–0,5)	0,5 (0,1–1,2)	0,8 (0,3–1,6)	0,7 (0,2–1,7)	0,2 (0–0,9)	2 (1–3)	0,2 (0–1,2)	0,5 (0,2 - 1)
**Jämtland/ Härjedalen**	0 (0–4,2)	1,6 (0,3–4,5)	1,9 (0,4–5,6)	1,7 (0,5–3,9)	3,2 (1,5–6)	1,2 (0,3–2,9)	1,1 (0,2–3,3)	0,8 (0 - 4,3)
**Jönköping**	0,2 (0–1,3)	0 (0–1,1)	0,3 (0–1,9)	0,4 (0,1–1,5)	0,4 (0,1–1,2)	1 (1–2)	1 (1–2)	0,3 (0 - 1,6)
**Kalmar**	0 (0–1,5)	0 (0–1,9)	0,4 (0–2)	0 (0–1,7)	0 (0–1,6)	1 (0,1–3,4)	0 (0–2,2)	0 (0 -0 ,7)
**Kronoberg**	1,2 (0,3–3)	2,7 (1,1–5,5)	1,4 (0,4–3,2)	10 (6–14)	2,9 (1,2–5,8)	0,5 (0–2,8)	0 (0–1,2)	0 (0 - 2,8)
**Norrbotten**	0 (0–0,7)	0 (0–1,7)	0 (0–0,8)	–	0 (0–2,1)	0,2 (0–0,9)	0 (0–7)	
**Skåne**	0 (0–1)	1 (0–1)	1 (0–1)	0,2 (0–0,6)	1 (1–2)	1 (1–2)	1 (0–1)	0,2 (0 - 0,5)
**Stockholm**	3 (2–5)	6 (4–8)	6 (4–9)	5 (4–8)	3 (2–5)	3 (2–3)	4 (3–5)	2 (2 - 3)
**Sörmland**	1,7 (0,8–3,2)	3 (1–5)	2 (1–3)	0,8 (0,2–2)	0,9 (0,3–2,1)	0,8 (0,3–1,6)	3 (2–4)	0,7 (0,3 - 1,5

We combine this data to estimate the number of cases due to the original strain in the matlab code Wuhan_strain_data.m. We do not have a fixed number for the percentage of alpha in the cases around week 17, which is the end of the time window we work with, but the below graph from the PHA https://www.folkhalsomyndigheten.se/smittskydd-beredskap/utbrott/aktuella-utbrott/covid-19/statistik-och-analyser/sars-cov-2-virusvarianter-av-sarskild-betydelse/ indicates that alpha is completely dominating and that this time point is just before delta starts to take over. We there for interpolate the cases caused by the original strain from the last available data point in week 13 to 0 in week 17.

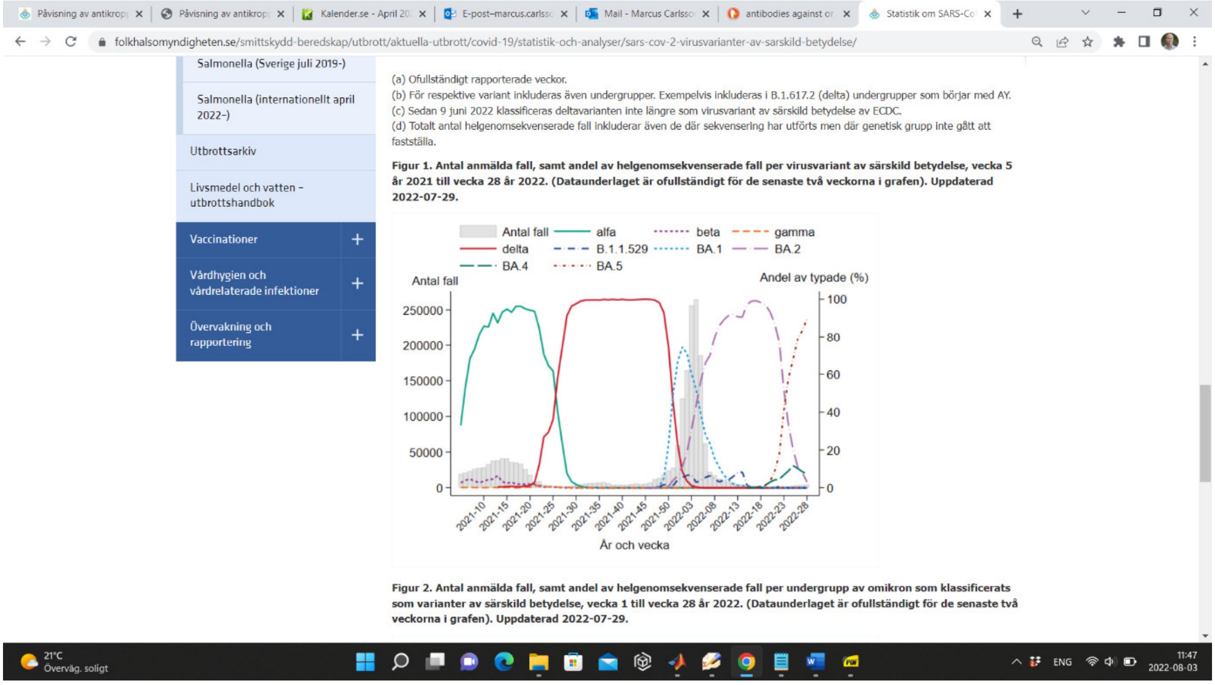

For the amount of vaccinated we relied on the file Folkhalsomyndigheten_Covid19_Vaccine.xlxs, which (at the time of writing) is freely downloadable from the Swedish Public Health Agency homepage https://www.folkhalsomyndigheten.se/smittskydd-beredskap/utbrott/aktuella-utbrott/covid-19/statistik-och-analyser/statistik-over-registrerade-vaccinationer-covid-19/

We use week data on amount of two shot vaccinations given in stockholm county. We have gathered the relevant data for easy access in the screenshots below:

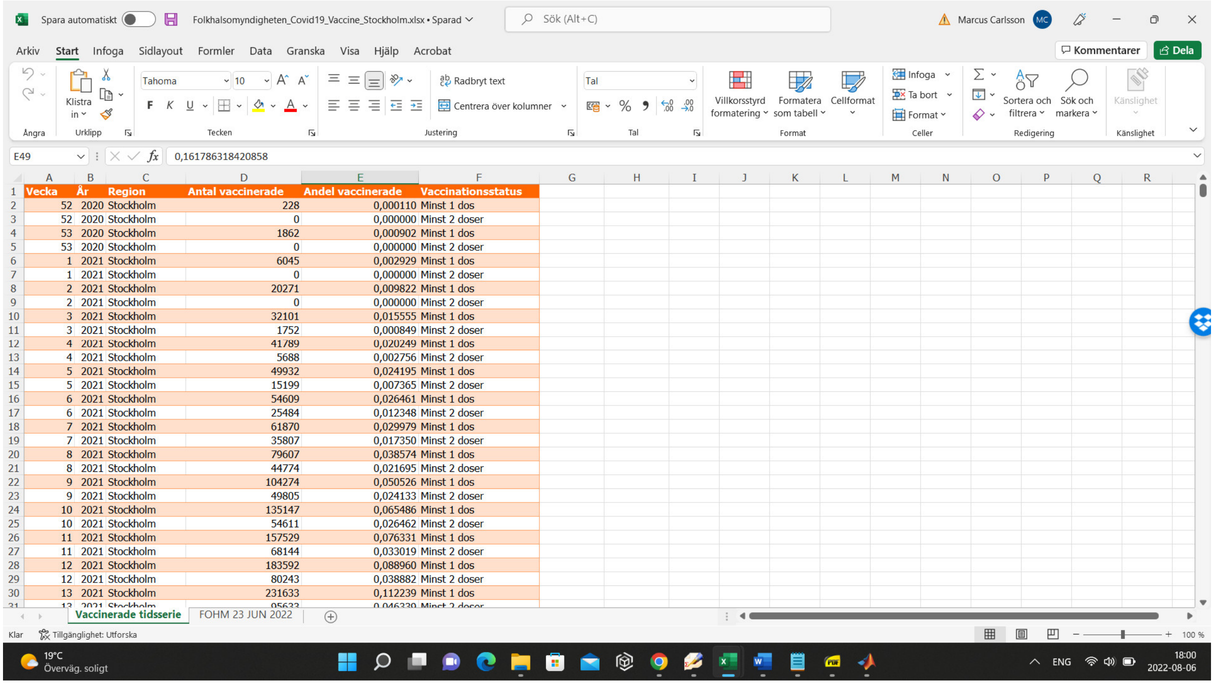

Vecka	År	Region	Antal vaccinerade	Andel vaccinerade	Vaccinationsstatus
52	2020	Stockholm	228	0,000110	Minst 1 dos
52	2020	Stockholm	0	0,000000	Minst 2 doser
53	2020	Stockholm	1862	0,000902	Minst 1 dos
53	2020	Stockholm	0	0,000000	Minst 2 doser
1	2021	Stockholm	6045	0,002929	Minst 1 dos
1	2021	Stockholm	0	0,000000	Minst 2 doser
2	2021	Stockholm	20271	0,009822	Minst 1 dos
2	2021	Stockholm	0	0,000000	Minst 2 doser
3	2021	Stockholm	32101	0,015555	Minst 1 dos
3	2021	Stockholm	1752	0,000849	Minst 2 doser
4	2021	Stockholm	41789	0,020249	Minst 1 dos
4	2021	Stockholm	5688	0,002756	Minst 2 doser
5	2021	Stockholm	49932	0,024195	Minst 1 dos
5	2021	Stockholm	15199	0,007365	Minst 2 doser
6	2021	Stockholm	54609	0,026461	Minst 1 dos
6	2021	Stockholm	25484	0,012348	Minst 2 doser
7	2021	Stockholm	61870	0,029979	Minst 1 dos
7	2021	Stockholm	35807	0,017350	Minst 2 doser
8	2021	Stockholm	79607	0,038574	Minst 1 dos
8	2021	Stockholm	44774	0,021695	Minst 2 doser
9	2021	Stockholm	104274	0,050526	Minst 1 dos
9	2021	Stockholm	49805	0,024133	Minst 2 doser
10	2021	Stockholm	135147	0,065486	Minst 1 dos
10	2021	Stockholm	54611	0,026462	Minst 2 doser
11	2021	Stockholm	157529	0,076331	Minst 1 dos
11	2021	Stockholm	68144	0,033019	Minst 2 doser
12	2021	Stockholm	183592	0,088960	Minst 1 dos
12	2021	Stockholm	80243	0,038882	Minst 2 doser
13	2021	Stockholm	231633	0,112239	Minst 1 dos

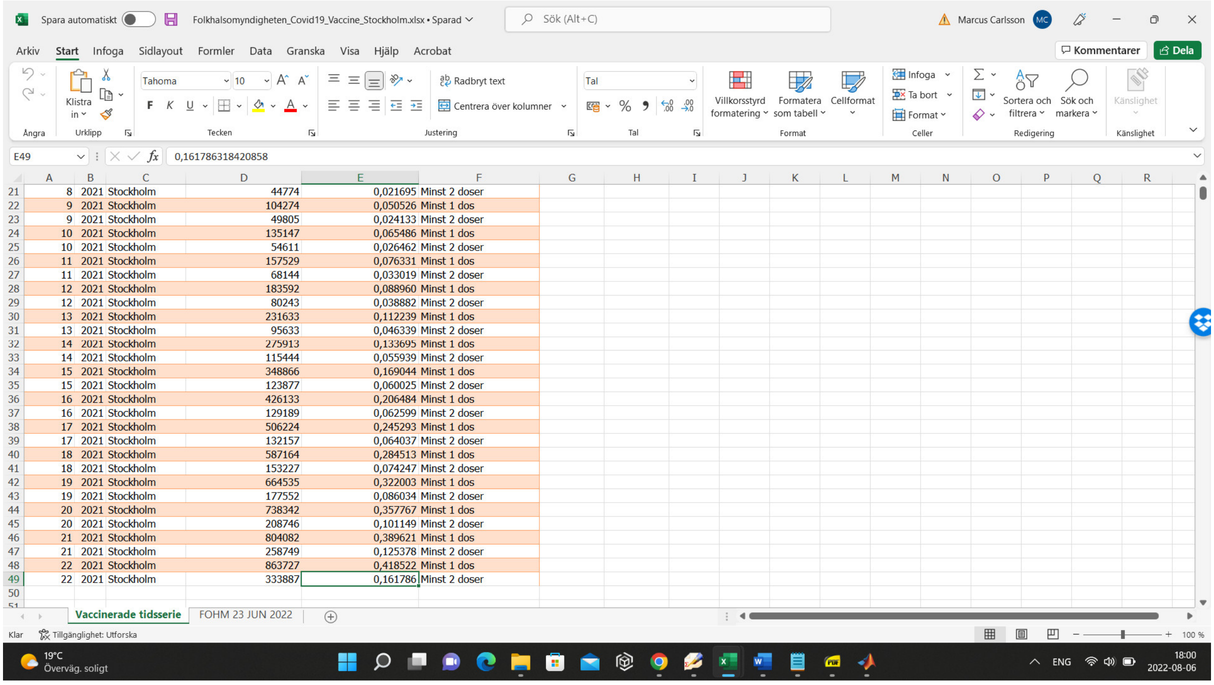

8	2021	Stockholm	44774	0,021695	Minst 2 doser
9	2021	Stockholm	104274	0,050526	Minst 1 dos
9	2021	Stockholm	49805	0,024133	Minst 2 doser
10	2021	Stockholm	135147	0,065486	Minst 1 dos
10	2021	Stockholm	54611	0,026462	Minst 2 doser
11	2021	Stockholm	157529	0,076331	Minst 1 dos
11	2021	Stockholm	68144	0,033019	Minst 2 doser
12	2021	Stockholm	183592	0,088960	Minst 1 dos
12	2021	Stockholm	80243	0,038882	Minst 2 doser
13	2021	Stockholm	231633	0,112239	Minst 1 dos
13	2021	Stockholm	95633	0,046339	Minst 2 doser
14	2021	Stockholm	275913	0,133695	Minst 1 dos
14	2021	Stockholm	115444	0,055939	Minst 2 doser
15	2021	Stockholm	348866	0,169044	Minst 1 dos
15	2021	Stockholm	123877	0,060025	Minst 2 doser
16	2021	Stockholm	426133	0,206484	Minst 1 dos
16	2021	Stockholm	129189	0,062599	Minst 2 doser
17	2021	Stockholm	506224	0,245293	Minst 1 dos
17	2021	Stockholm	132157	0,064037	Minst 2 doser
18	2021	Stockholm	587164	0,284513	Minst 1 dos
18	2021	Stockholm	153227	0,074247	Minst 2 doser
19	2021	Stockholm	664535	0,322003	Minst 1 dos
19	2021	Stockholm	177552	0,086034	Minst 2 doser
20	2021	Stockholm	738342	0,357767	Minst 1 dos
20	2021	Stockholm	208746	0,101149	Minst 2 doser
21	2021	Stockholm	804082	0,389621	Minst 1 dos
21	2021	Stockholm	258749	0,125378	Minst 2 doser
22	2021	Stockholm	863727	0,418522	Minst 1 dos
22	2021	Stockholm	333887	0,161786	Minst 2 doser

Finally, for data on seroprevalence of antibodies for SARS-CoV-2 we rely on blood donor sampling performed at various timepoints by the public health agency, which is reported in the document Påvisning av antikroppar efter genomgången COVID-19 i blodprov från öppenvården found at the link https://www.folkhalsomyndigheten.se/contentassets/9c5893f84bd049e691562b9eeb0ca280/pavisning-antikroppar-mot-sars-cov-2-blodprov-oppenvarden.pdf

The data-points mentioned in the paper are highlighted in yellow in the below screenshots from the above mentioned document. Data points concerning country wide seroprevalence in April:

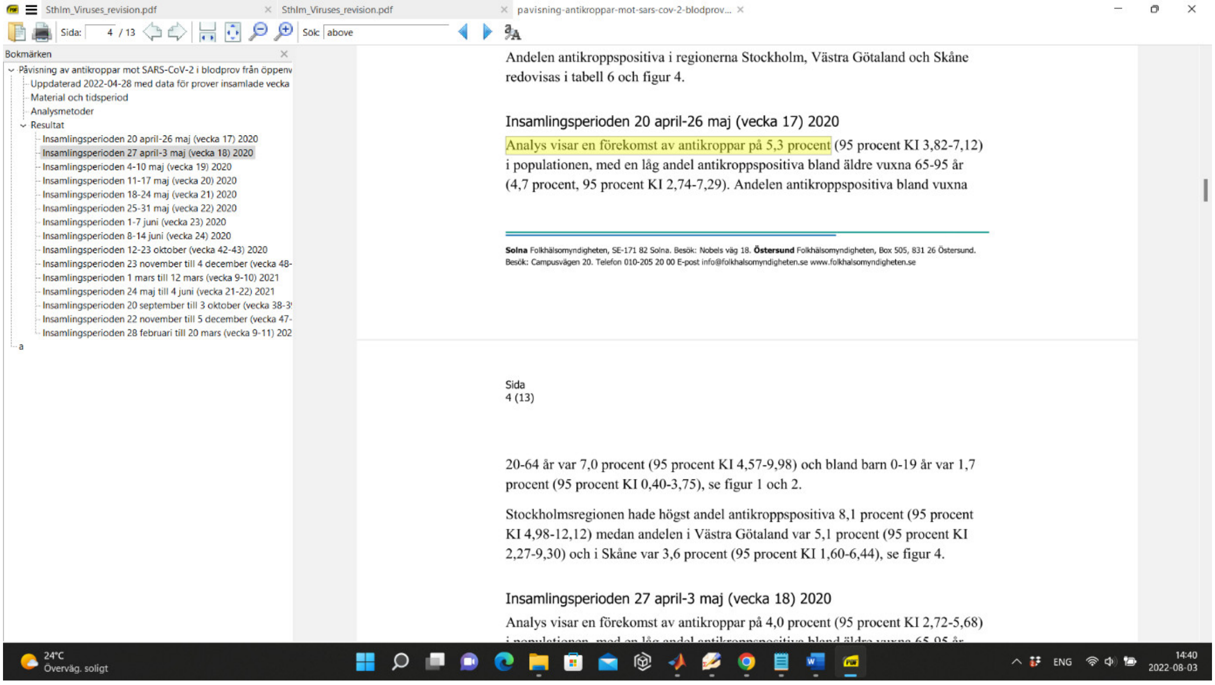

Andelen antikroppspositiva i regionerna Stockholm, Västra Götaland och Skåne redovisas i tabell 6 och figur 4.

Insamlingsperioden 20 april-26 maj (vecka 17) 2020

Analys visar en förekomst av antikroppar på 5,3 procent (95 procent KI 3,82-7,12) i populationen, med en låg andel antikroppspositiva bland äldre vuxna 65-95 år (4,7 procent, 95 procent KI 2,74-7,29). Andelen antikroppspositiva bland vuxna

Solna Folkhälsomyndigheten, SE-171 82 Solna. Besök: Nobels väg 18. **Östersund** Folkhälsomyndigheten, Box 505, 831 26 Östersund. Besök: Campusvägen 20. Telefon 010-205 20 00 E-post info@folkhalsomyndigheten.se www.folkhalsomyndigheten.se

Sida
4 (13)

20-64 år var 7,0 procent (95 procent KI 4,57-9,98) och bland barn 0-19 år var 1,7 procent (95 procent KI 0,40-3,75), se figur 1 och 2.

Stockholmsregionen hade högst andel antikroppspositiva 8,1 procent (95 procent KI 4,98-12,12) medan andelen i Västra Götaland var 5,1 procent (95 procent KI 2,27-9,30) och i Skåne var 3,6 procent (95 procent KI 1,60-6,44), se figur 4.

Insamlingsperioden 27 april-3 maj (vecka 18) 2020

Analys visar en förekomst av antikroppar på 4,0 procent (95 procent KI 2,72-5,68) [illegible]

Data points concerning seroprevalence in Stockholm County:

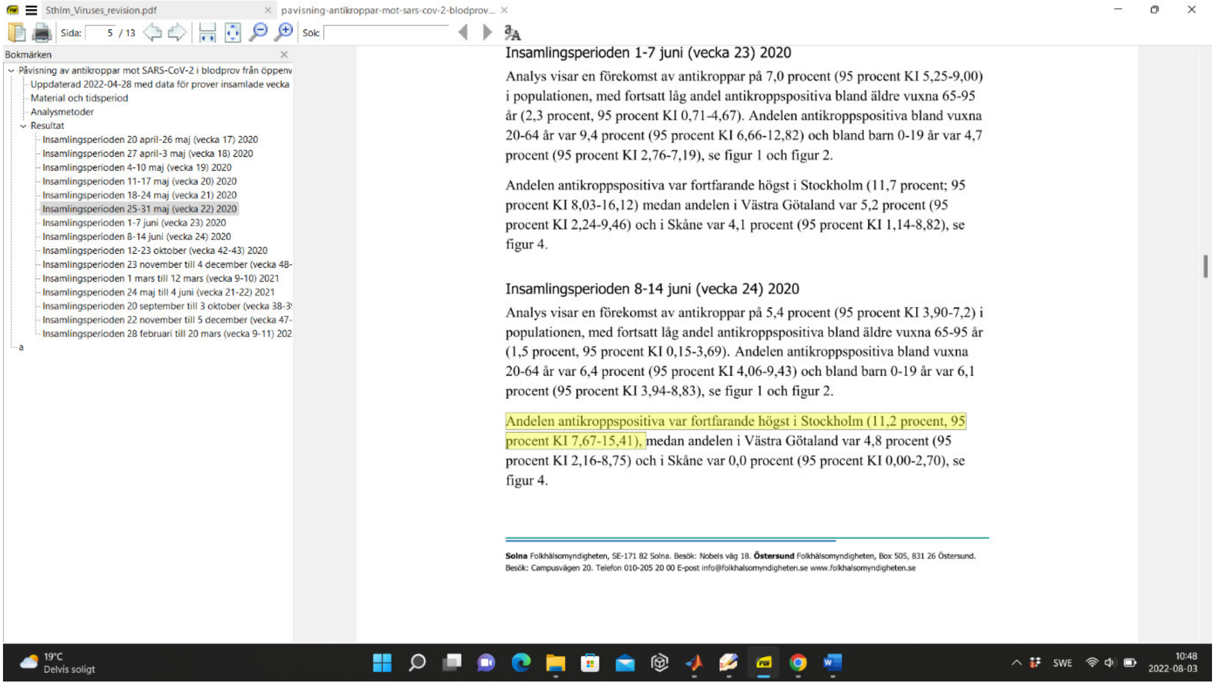

Insamlingsperioden 1-7 juni (vecka 23) 2020

Analys visar en förekomst av antikroppar på 7,0 procent (95 procent KI 5,25-9,00) i populationen, med fortsatt låg andel antikroppspositiva bland äldre vuxna 65-95 år (2,3 procent, 95 procent KI 0,71-4,67). Andelen antikroppspositiva bland vuxna 20-64 år var 9,4 procent (95 procent KI 6,66-12,82) och bland barn 0-19 år var 4,7 procent (95 procent KI 2,76-7,19), se figur 1 och figur 2.

Andelen antikroppspositiva var fortfarande högst i Stockholm (11,7 procent; 95 procent KI 8,03-16,12) medan andelen i Västra Götaland var 5,2 procent (95 procent KI 2,24-9,46) och i Skåne var 4,1 procent (95 procent KI 1,14-8,82), se figur 4.

Insamlingsperioden 8-14 juni (vecka 24) 2020

Analys visar en förekomst av antikroppar på 5,4 procent (95 procent KI 3,90-7,2) i populationen, med fortsatt låg andel antikroppspositiva bland äldre vuxna 65-95 år (1,5 procent, 95 procent KI 0,15-3,69). Andelen antikroppspositiva bland vuxna 20-64 år var 6,4 procent (95 procent KI 4,06-9,43) och bland barn 0-19 år var 6,1 procent (95 procent KI 3,94-8,83), se figur 1 och figur 2.

Andelen antikroppspositiva var fortfarande högst i Stockholm (11,2 procent, 95 procent KI 7,67-15,41), medan andelen i Västra Götaland var 4,8 procent (95 procent KI 2,16-8,75) och i Skåne var 0,0 procent (95 procent KI 0,00-2,70), se figur 4.

Solna Folkhälsomyndigheten, SE-171 82 Solna. Besök: Nobels väg 18. **Östersund** Folkhälsomyndigheten, Box 505, 831 26 Östersund. Besök: Campusvägen 20. Telefon 010-205 20 00 E-post info@folkhalsomyndigheten.se www.folkhalsomyndigheten.se

Bokmärken
Påvisning av antikroppar mot SARS-CoV-2 i blodprov från öppenv
Uppdaterad 2022-04-28 med data för prover insamlade vecka
Material och tidsperiod
Analysmetoder
Resultat
Insamlingsperioden 20 april-26 maj (vecka 17) 2020
Insamlingsperioden 27 april-3 maj (vecka 18) 2020
Insamlingsperioden 4-10 maj (vecka 19) 2020
Insamlingsperioden 11-17 maj (vecka 20) 2020
Insamlingsperioden 18-24 maj (vecka 21) 2020
Insamlingsperioden 25-31 maj (vecka 22) 2020
Insamlingsperioden 1-7 juni (vecka 23) 2020
Insamlingsperioden 8-14 juni (vecka 24) 2020
Insamlingsperioden 12-23 oktober (vecka 42-43) 2020
Insamlingsperioden 23 november till 4 december (vecka 48-
Insamlingsperioden 1 mars till 12 mars (vecka 9-10) 2021
Insamlingsperioden 24 maj till 4 juni (vecka 21-22) 2021
Insamlingsperioden 20 september till 3 oktober (vecka 38-3
Insamlingsperioden 22 november till 5 december (vecka 47-
Insamlingsperioden 28 februari till 20 mars (vecka 9-11) 202

Insamlingsperioden 12-23 oktober (vecka 42-43) 2020

Analys visar en förekomst av antikroppar på 6,7 procent (95 procent KI 5,73-7,85) i populationen, med fortsatt låg andel antikroppspositiva bland äldre vuxna 65-95 år (1,9 procent, 95 procent KI 1,19-2,85). Andelen antikroppspositiva bland vuxna 20-64 år var 8,6 procent (95 procent KI 7,00-10,47). Bland barn och ungdomar 0-19 år var andelen antikroppspositiva 5,9 procent (95 procent KI 4,65-7,41). se figur 1 och figur 2.

Andelen antikroppspositiva bland barn 0-11 år var 4,6 procent (95 procent KI 2,98-6,67). Bland barn 12-15 år var andelen med antikroppar 5,6 procent (95 procent KI 3,31-8,68), medan den bland ungdomar 16-19 år var 9,3 procent (95 procent KI 6,17-13,31), se figur 3.

Andelen antikroppspositiva var fortfarande högst i Stockholm (9,8 procent; 95 procent KI 7,80-12,11), medan andelen i Västra Götaland var 8,8 procent (95 procent KI 6,61-11,44) och i Skåne var 1,0 procent (95 procent KI 0,11-2,37), se figur 4.

Andelen antikroppspositiva i övriga undersökta regioner var 6,1 procent (95 procent KI 3,32-9,89) i Uppsala, 6,0 procent (95 procent KI 2,09-12,15) i Örebro, 5,3 procent (95 procent KI 1,05-13,15) i Kalmar och 0,5 procent (95 procent KI 0,00-6,04) i Västerbotten.

Insamlingsperioden 1 mars till 12 mars (vecka 9-10) 2021

Analys visar en förekomst av antikroppar på 20,9 procent (95 procent KI 19,63-22,30) i populationen, med fortsatt lägst andel antikroppspositiva bland äldre vuxna 65-95 år (15,0 procent, 95 procent KI 12,82-17,30). Andelen antikroppspositiva bland vuxna 20-64 år var 21,9 procent (95 procent KI 19,86-23,96). Bland barn och ungdomar 0-19 år var andelen antikroppspositiva 23,4 procent (95 procent KI 21,36-25,49), se figur 1 och figur 2.

Andelen antikropps positiva bland barn 0-11 år var 20,2 procent (95 procent KI 17,53-23,15). Bland barn 12-15 år var andelen med antikroppar 24,7 procent (95 procent KI 20,84-28,77), medan den bland ungdomar 16-19 år var 29,5 procent (95 procent KI 24,60-34,68), se figur 3.

Andelen antikroppspositiva i Stockholm var 22,8 procent (95 procent KI 20,68-24,99), medan andelen i Västra Götaland var 20,8 procent (95 procent KI 17,76-24,16) och i Skåne var 20,3 procent (95 procent KI 17,67-23,14), se figur 4.

Andelen antikroppspositiva i övriga undersökta regioner var 19,7 procent (95 procent KI 14,69-25,47) i Jönköping, 17,0 procent (95 procent KI 11,32-23,87) i Kalmar, 17,5 procent (95 procent KI 12,27-23,64) i Örebro och 16,8 procent (95 procent KI 11,50-23,09) i Västerbotten.

Insamlingsperioden 24 maj till 4 juni (vecka 21-22) 2021

Analys visar en förekomst av antikroppar på 52,7 procent (95 procent KI 50,91-54,57) i populationen. Högst andel antikroppspositiva var bland äldre vuxna 65-95 år (85,0 procent, 95 procent KI 82,47-87,25). Andelen antikroppspositiva bland vuxna 20-64 år var 52,0 procent (95 procent KI 49,18-54,89). Bland barn och ungdomar 0-19 år var andelen antikroppspositiva 29,7 procent (95 procent KI 27,41-32,07), se figur 1 och figur 2.

Andelen antikroppspositiva bland barn 0-11 år var 23,3 procent (95 procent KI 20,29-26,45). Bland barn 12-15 år var andelen med antikroppar 34,9 procent (95

Data points concerning children (countrywide)

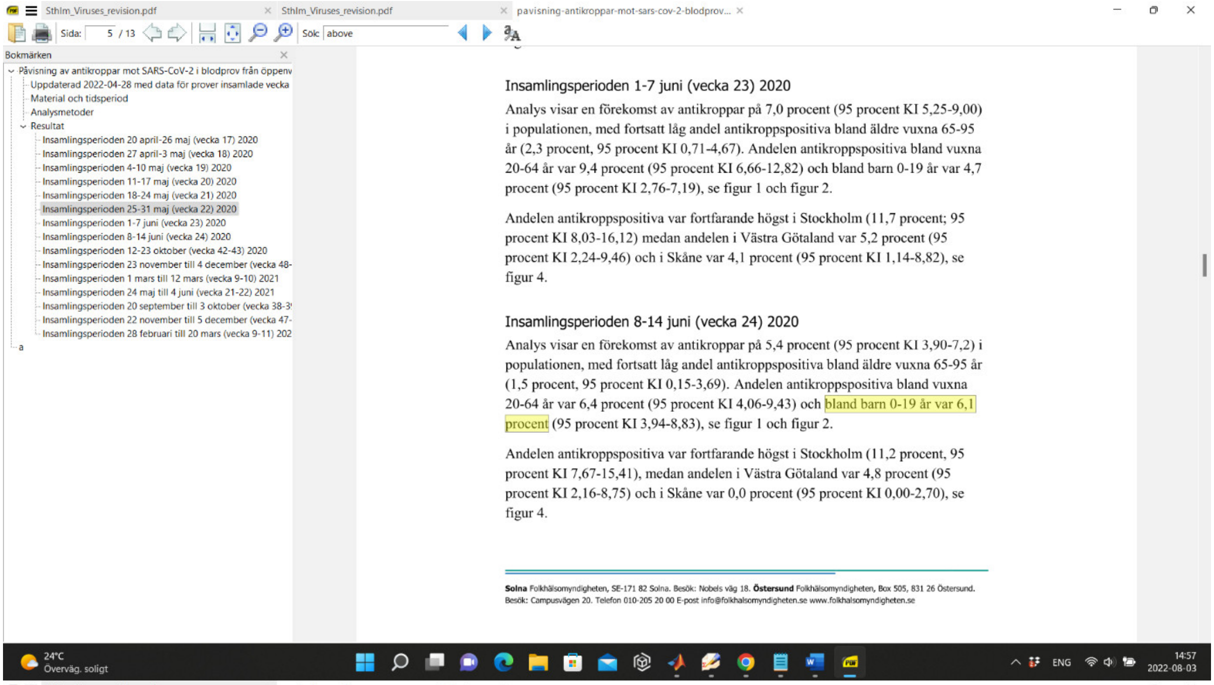

Insamlingsperioden 1-7 juni (vecka 23) 2020

Analys visar en förekomst av antikroppar på 7,0 procent (95 procent KI 5,25-9,00) i populationen, med fortsatt låg andel antikroppspositiva bland äldre vuxna 65-95 år (2,3 procent, 95 procent KI 0,71-4,67). Andelen antikroppspositiva bland vuxna 20-64 år var 9,4 procent (95 procent KI 6,66-12,82) och bland barn 0-19 år var 4,7 procent (95 procent KI 2,76-7,19), se figur 1 och figur 2.

Andelen antikroppspositiva var fortfarande högst i Stockholm (11,7 procent; 95 procent KI 8,03-16,12) medan andelen i Västra Götaland var 5,2 procent (95 procent KI 2,24-9,46) och i Skåne var 4,1 procent (95 procent KI 1,14-8,82), se figur 4.

Insamlingsperioden 8-14 juni (vecka 24) 2020

Analys visar en förekomst av antikroppar på 5,4 procent (95 procent KI 3,90-7,2) i populationen, med fortsatt låg andel antikroppspositiva bland äldre vuxna 65-95 år (1,5 procent, 95 procent KI 0,15-3,69). Andelen antikroppspositiva bland vuxna 20-64 år var 6,4 procent (95 procent KI 4,06-9,43) och bland barn 0-19 år var 6,1 procent (95 procent KI 3,94-8,83), se figur 1 och figur 2.

Andelen antikroppspositiva var fortfarande högst i Stockholm (11,2 procent, 95 procent KI 7,67-15,41), medan andelen i Västra Götaland var 4,8 procent (95 procent KI 2,16-8,75) och i Skåne var 0,0 procent (95 procent KI 0,00-2,70), se figur 4.

Solna Folkhälsomyndigheten, SE-171 82 Solna. Besök: Nobels väg 18. **Östersund** Folkhälsomyndigheten, Box 505, 831 26 Östersund. Besök: Campusvägen 20. Telefon 010-205 20 00 E-post info@folkhalsomyndigheten.se www.folkhalsomyndigheten.se

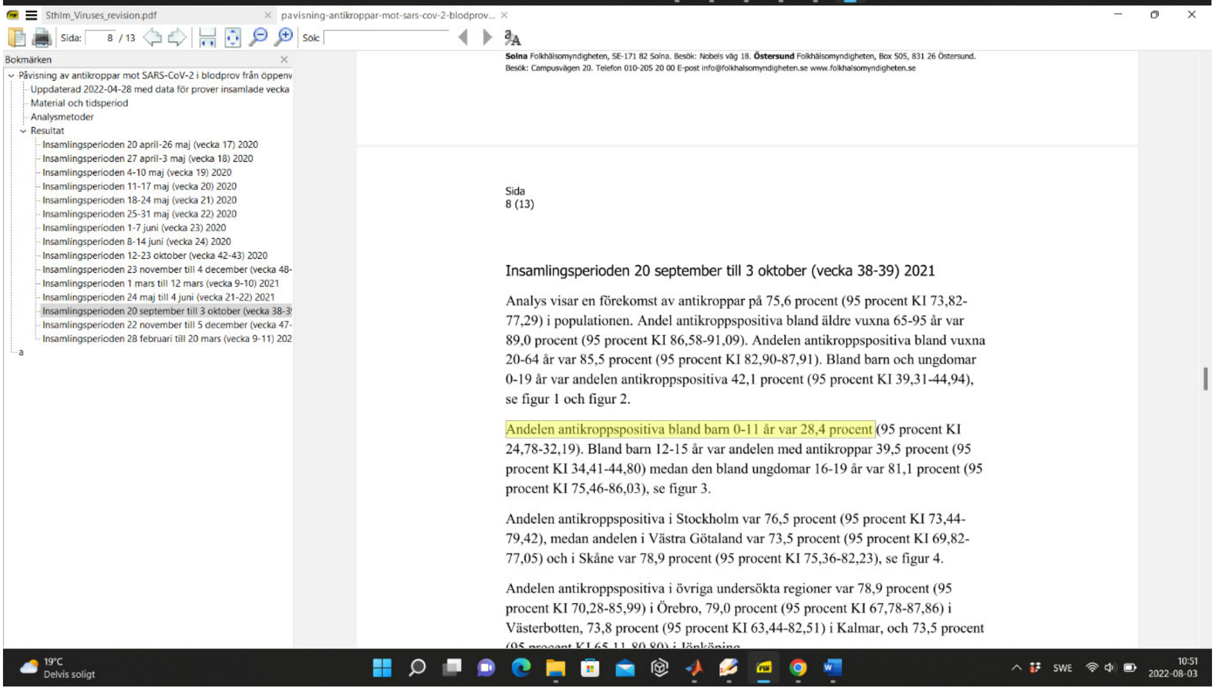

Solna Folkhälsomyndigheten, SE-171 82 Solna. Besök: Nobels väg 18. **Östersund** Folkhälsomyndigheten, Box 505, 831 26 Östersund. Besök: Campusvägen 20. Telefon 010-205 20 00 E-post info@folkhalsomyndigheten.se www.folkhalsomyndigheten.se

Sida
8 (13)

Insamlingsperioden 20 september till 3 oktober (vecka 38-39) 2021

Analys visar en förekomst av antikroppar på 75,6 procent (95 procent KI 73,82-77,29) i populationen. Andel antikroppspositiva bland äldre vuxna 65-95 år var 89,0 procent (95 procent KI 86,58-91,09). Andelen antikroppspositiva bland vuxna 20-64 år var 85,5 procent (95 procent KI 82,90-87,91). Bland barn och ungdomar 0-19 år var andelen antikroppspositiva 42,1 procent (95 procent KI 39,31-44,94), se figur 1 och figur 2.

Andelen antikroppspositiva bland barn 0-11 år var 28,4 procent (95 procent KI 24,78-32,19). Bland barn 12-15 år var andelen med antikroppar 39,5 procent (95 procent KI 34,41-44,80) medan den bland ungdomar 16-19 år var 81,1 procent (95 procent KI 75,46-86,03), se figur 3.

Andelen antikroppspositiva i Stockholm var 76,5 procent (95 procent KI 73,44-79,42), medan andelen i Västra Götaland var 73,5 procent (95 procent KI 69,82-77,05) och i Skåne var 78,9 procent (95 procent KI 75,36-82,23), se figur 4.

Andelen antikroppspositiva i övriga undersökta regioner var 78,9 procent (95 procent KI 70,28-85,99) i Örebro, 79,0 procent (95 procent KI 67,78-87,86) i Västerbotten, 73,8 procent (95 procent KI 63,44-82,51) i Kalmar, och 73,5 procent [illegible]

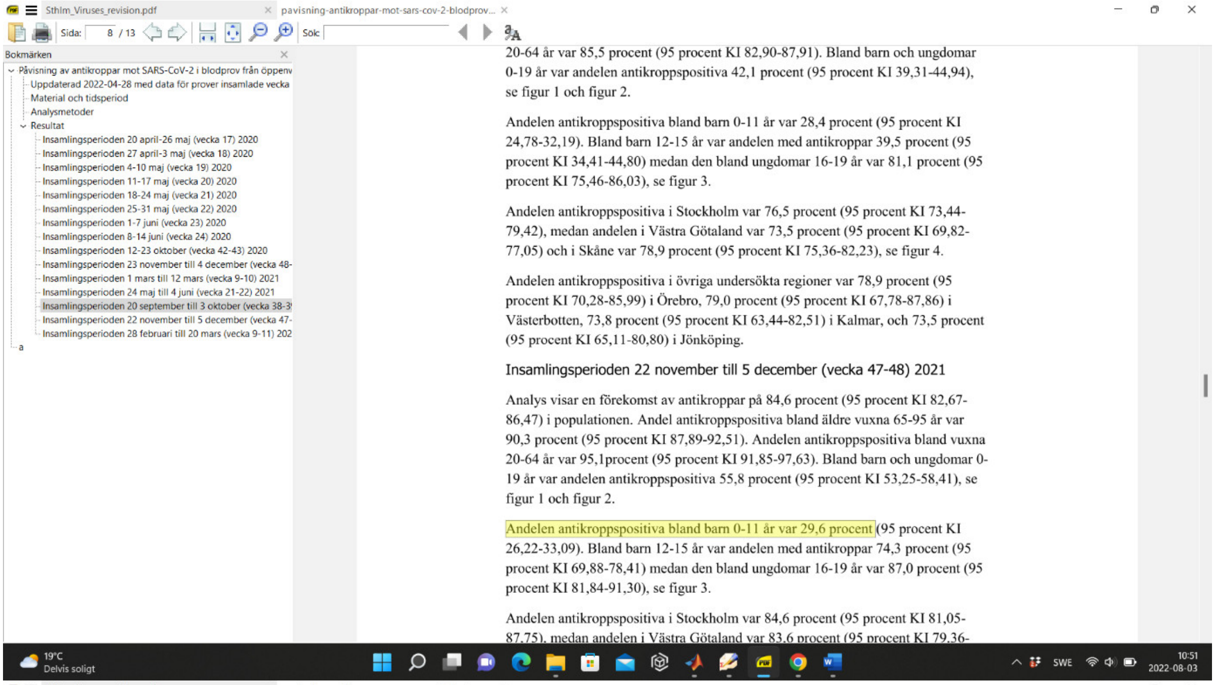

20-64 år var 85,5 procent (95 procent KI 82,90-87,91). Bland barn och ungdomar 0-19 år var andelen antikroppspositiva 42,1 procent (95 procent KI 39,31-44,94), se figur 1 och figur 2.

Andelen antikroppspositiva bland barn 0-11 år var 28,4 procent (95 procent KI 24,78-32,19). Bland barn 12-15 år var andelen med antikroppar 39,5 procent (95 procent KI 34,41-44,80) medan den bland ungdomar 16-19 år var 81,1 procent (95 procent KI 75,46-86,03), se figur 3.

Andelen antikroppspositiva i Stockholm var 76,5 procent (95 procent KI 73,44-79,42), medan andelen i Västra Götaland var 73,5 procent (95 procent KI 69,82-77,05) och i Skåne var 78,9 procent (95 procent KI 75,36-82,23), se figur 4.

Andelen antikroppspositiva i övriga undersökta regioner var 78,9 procent (95 procent KI 70,28-85,99) i Örebro, 79,0 procent (95 procent KI 67,78-87,86) i Västerbotten, 73,8 procent (95 procent KI 63,44-82,51) i Kalmar, och 73,5 procent (95 procent KI 65,11-80,80) i Jönköping.

Insamlingsperioden 22 november till 5 december (vecka 47-48) 2021

Analys visar en förekomst av antikroppar på 84,6 procent (95 procent KI 82,67-86,47) i populationen. Andel antikroppspositiva bland äldre vuxna 65-95 år var 90,3 procent (95 procent KI 87,89-92,51). Andelen antikroppspositiva bland vuxna 20-64 år var 95,1procent (95 procent KI 91,85-97,63). Bland barn och ungdomar 0-19 år var andelen antikroppspositiva 55,8 procent (95 procent KI 53,25-58,41), se figur 1 och figur 2.

Andelen antikroppspositiva bland barn 0-11 år var 29,6 procent (95 procent KI 26,22-33,09). Bland barn 12-15 år var andelen med antikroppar 74,3 procent (95 procent KI 69,88-78,41) medan den bland ungdomar 16-19 år var 87,0 procent (95 procent KI 81,84-91,30), se figur 3.

Andelen antikroppspositiva i Stockholm var 84,6 procent (95 procent KI 81,05-87,75), medan andelen i Västra Götaland var 83,6 procent (95 procent KI 79,36-

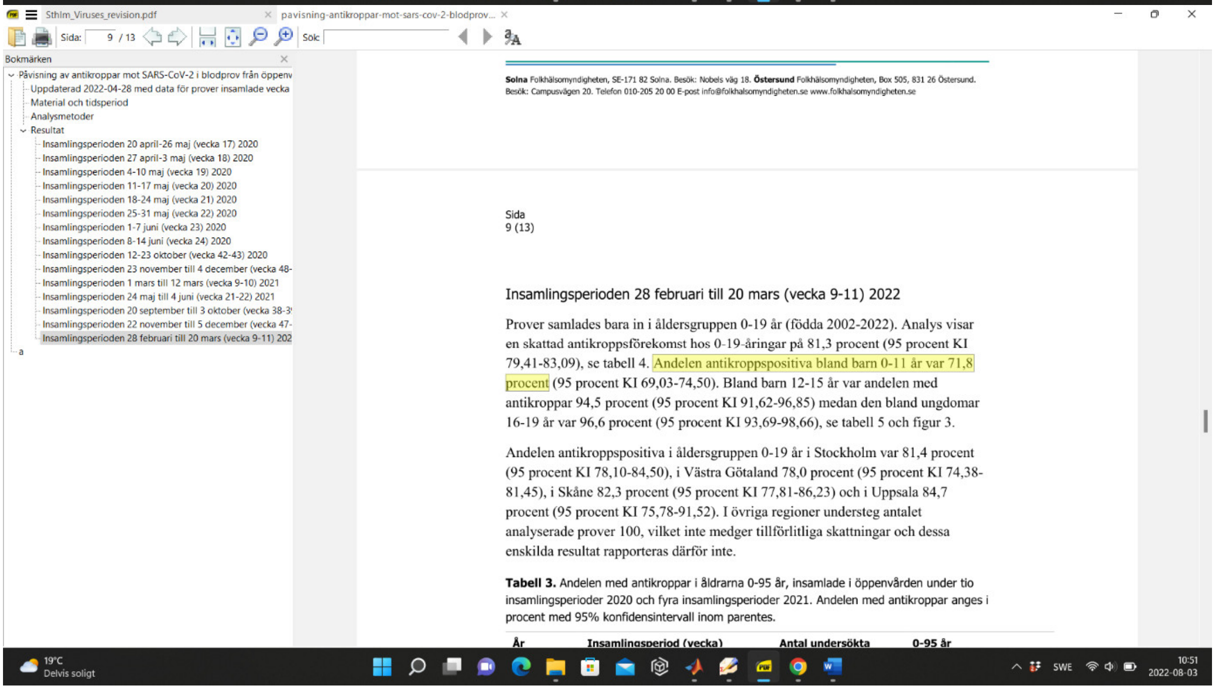

Solna Folkhälsomyndigheten, SE-171 82 Solna. Besök: Nobels väg 18. Östersund Folkhälsomyndigheten, Box 505, 831 26 Östersund. Besök: Campusvägen 20. Telefon 010-205 20 00 E-post info@folkhalsomyndigheten.se www.folkhalsomyndigheten.se

Sida
9 (13)

Insamlingsperioden 28 februari till 20 mars (vecka 9-11) 2022

Prover samlades bara in i åldersgruppen 0-19 år (födda 2002-2022). Analys visar en skattad antikroppsförekomst hos 0-19-åringar på 81,3 procent (95 procent KI 79,41-83,09), se tabell 4. Andelen antikroppspositiva bland barn 0-11 år var 71,8 procent (95 procent KI 69,03-74,50). Bland barn 12-15 år var andelen med antikroppar 94,5 procent (95 procent KI 91,62-96,85) medan den bland ungdomar 16-19 år var 96,6 procent (95 procent KI 93,69-98,66), se tabell 5 och figur 3.

Andelen antikroppspositiva i åldersgruppen 0-19 år i Stockholm var 81,4 procent (95 procent KI 78,10-84,50), i Västra Götaland 78,0 procent (95 procent KI 74,38-81,45), i Skåne 82,3 procent (95 procent KI 77,81-86,23) och i Uppsala 84,7 procent (95 procent KI 75,78-91,52). I övriga regioner understeg antalet analyserade prover 100, vilket inte medger tillförlitliga skattningar och dessa enskilda resultat rapporteras därför inte.

Tabell 3. Andelen med antikroppar i åldrarna 0-95 år, insamlade i öppenvården under tio insamlingsperioder 2020 och fyra insamlingsperioder 2021. Andelen med antikroppar anges i procent med 95% konfidensintervall inom parentes.

År	Insamlingsperiod (vecka)	Antal undersökta	0-95 år

Finally, Google-mobility data has been downloaded from https://www.google.com/covid19/mobility/ on 2022-08-06 and the relevant data for Sweden is found in Google_mobility_Stockholm_20200215_to_20220727.mat on the GitHub-page
